# Identification of Shared Molecular Signatures Indicate the Susceptibility of Endometriosis to Multiple Sclerosis

**DOI:** 10.3389/fgene.2018.00042

**Published:** 2018-02-16

**Authors:** Amit Katiyar, Sujata Sharma, Tej P. Singh, Punit Kaur

**Affiliations:** Department of Biophysics, All India Institute of Medical Sciences, New Delhi, India

**Keywords:** endometriosis, multiple sclerosis, pathway analysis, enrichment analyses, autoimmune disease, immunodeficiency, meta-analysis

## Abstract

Women with endometriosis (EMS) appear to be at a higher risk of developing other autoimmune diseases predominantly multiple sclerosis (MS). Though EMS and MS are evidently diverse in their phenotype, they are linked by a common autoimmune condition or immunodeficiency which could play a role in the expansion of endometriosis and possibly increase the risk of developing MS in women with EMS. However, the common molecular links connecting EMS with MS are still unclear. We conducted a meta-analysis of microarray experiments focused on EMS and MS with their respective controls. The GEO2R web application discovered a total of 711 and 1516 genes that are differentially expressed across the experimental conditions in EMS and MS, respectively with 129 shared DEGs between them. The functional enrichment analysis of DEGs predicts the shared gene expression signatures as well as the overlapping biological processes likely to infer the co-occurrence of EMS with MS. Network based meta-analysis unveiled six interaction networks/crosstalks through overlapping edges between commonly dysregulated pathways of EMS and MS. The PTPN1, ERBB3, and CDH1 were observed to be the highly ranked hub genes connected with disease-related genes of both EMS and MS. Androgen receptor (AR) and nuclear factor-kB p65 (RelA) were observed to be the most enriched transcription factor in the upstream of shared down-regulated and up-regulated genes, respectively. The two disease sample sets compared through crosstalk interactions between shared pathways revealed commonly up- and down-regulated expressions of 10 immunomodulatory proteins as probable linkers between EMS and MS. This study pinpoints the number of shared genes, pathways, protein kinases, and upstream regulators that may help in the development of biomarkers for diagnosis of MS and endometriosis at the same time through improved understanding of shared molecular signatures and crosstalk.

## Introduction

Endometriosis (EMS) is an estrogen-dependent inflammatory disorder which affects approximately 5–10% of women in the reproductive age worldwide (Bulun, 2009). The endometrial tissue which normally is present inside the uterus is displaced outside in patients suffering from EMS resulting in pelvic pain and infertility (Capobianco and Rovere-Querini, 2013). Immunological factors are known to contribute significantly to the pathogenesis and pathophysiology of endometriosis (Berkkanoglu and Arici, 2003; Podgaec et al., 2007, 2010; Fairbanks et al., 2009; Nielsen et al., 2011; Capobianco and Rovere-Querini, 2013). The prime regulators of the innate immune response are macrophages which come into play in case of injury, damage and infection. Macrophages possess functionally diverse contrasting roles, as on one hand, they play a protective role through differentiation and regeneration of cells while on the other hand they stimulate the immune response leading to destruction of infected cells (Vogel et al., 2013). Pro-inflammatory cytokine (interferon-γ) activated macrophages are known to have an essential role in the onset and progression of endometriosis. The macrophages misinterpret the displaced ectopic endometrial tissue as an injury and hence, instead of removing the endometrial cells, they activate pathways that repair and enhance their survival leading to sustained endometrial tissue (Podgaec et al., 2010; Capobianco and Rovere-Querini, 2013). It has been reported that the women suffering from EMS are more prone to acquire other inflammatory autoimmune disorders especially multiple sclerosis (MS) (Nielsen et al., 2011; Mormile and Vittori, 2014). Multiple sclerosis (MS) is a chronic neuroinflammatory autoimmune disease of the central nervous system associated with neurodegeneration (Hickey, 1999; Compston and Coles, 2002; Szczucinski and Losy, 2007). Like endometriosis, macrophages have been observed to be directly associated in the pathogenesis of MS (Oreja-Guevara et al., 2012; Vogel et al., 2013). Additionally, T-helper 1 (Th1)/T-helper 2 (Th2) imbalance has been associated with both EMS and MS wherein the pro-inflammatory Th1 profile dominates over the Th2 anti-inflammatory response. This is similar to other autoimmune diseases where the immune system launches an attack on its own cells and tissues (Trapp et al., 1998; Peterson et al., 2001; Diestel et al., 2003; Aktas et al., 2005). Among the increased Th1 cytokines, it has been reported that Interferon-γ (IFN-γ) is strongly associated with the pathomechanisms of MS (Oreja-Guevara et al., 2012; Vogel et al., 2013). Though the association between these two heterogeneous diseases EMS and MS, is not clearly recognized, it may be attributable to differential gene expression and sharing of common dysregulated pathogenic pathways involved in the development of both diseases. A 'crosstalk' event between two pathways, thus, elucidates how one or more components of one pathway affect another through interactions with shared components, protein-protein interactions and transcriptional regulations (Lu et al., 2007; Guo and Wang, 2009; Housden and Perrimon, 2014). Therefore, an examination of possible crosstalks and shared components among common dysregulated pathways together with associated genes in both endometriosis and MS may be able to assist in the understanding of the disease mechanism.

Over the last two decades, a meta-analysis approach has been well exploited to uncover the shared molecular signatures between related diseases by integrating the publicly accessible microarray datasets (Silva et al., 2007; Higgs et al., 2012; Tuller et al., 2013; Jha et al., 2016). Recent studies have focused on identifying crosstalk among dysregulated pathways using expression profiles of genes from control vs. disease samples (Zhang et al., 2013; Niu et al., 2014, 2015; Chen et al., 2015). In the present study, we aimed to identify commonly dysregulated genes and pathways which probably co-occur in both EMS and MS to elucidate the relationship between the two diseases. We performed here a meta-analysis using gene expression data from microarray experiments of EMS and MS with their respective controls to predict the Differentially Expressed Genes (DEGs) involved in the respective diseases (Arasappan et al., 2011; Taminau et al., 2014). Widely used enrichment analysis methods such as Kyoto Encyclopedia of Genes and Genomes (KEGG), Gene Ontology (GO), and Protein-Protein Interactions (PPI) were adopted for the prediction of dysregulated pathways and subsequent possible crosstalk between EMS and MS. The findings from this study increase our understanding of the molecular mechanisms affecting both EMS and MS. Moreover, it brings forth the commonly shared genes, molecules and pathways co-existing in both EMS and MS which may be further explored as newer therapeutic targets.

## Materials and methods

### Data acquisition

Widely accessible gene expression datasets related to endometriosis (EMS) and MS were obtained from the Gene Expression Omnibus (GEO) database of NCBI (http://www.ncbi.nlm.nih.gov/geo/; Barrett and Edgar, 2006; Barrett et al., 2013). The keywords “endometriosis” and “multiple sclerosis” with “homo sapiens” or “human” were employed to mine the dataset. Studies evaluated on Affymetrix human gene expression dataset (irrespective of platform) containing samples from both normal and diseased tissue (more or less equally distributed) of women were taken. It was also ensured that these studies included only tissue samples that were not cultured *in vitro*. Similarly, tissue samples treated with any drugs before extraction were also excluded. The expression profiles of both primary and secondary cell cultures were also not considered for this analysis. Overall 14 datasets (7 each for EMS and MS) which met these criteria were selected from published studies and downloaded from GEO database. The expression datasets of EMS combined tissue samples from 7 GEO profiles, i.e., GSE11691, GSE25628, GSE51981, GSE6364, GSE7305, GSE7307, and GSE7846 were selected. Likewise, expression datasets of MS involved tissue samples from 7 GEO profiles, i.e., GSE16461, GSE21942, GSE26484, GSE38010, GSE41848, GSE41849, and GSE41890. These samples were further separated into groups according to tissue source. Datasets were not subjected to any additional normalization, as all the data obtained had already been processed/normalized and were cross-comparable. The related information regarding the dataset pertaining to the microarray platform used, sample type and sample size are listed in Table 1.

**Table 1:** Published datasets related to endometriosis **(A)** and multiple sclerosis **(B)** used in this study.

**GEO accession**	**Platform**	**No. of probes**	**No. of samples (control/disease)**	**Tissue stage**	**References**
**(A) ENDOMETRIOSIS (EMS)**					
GSE7305	GPL570: [HG-U133_Plus_2]	54675	20 (10/10)	Follicular phase/Ovarian-Follicular phase	Hever et al., 2007
GSE7307	GPL570: [HG-U133_Plus_2]	54675	41 (23/18)	Follicular phase/Ovarian-Follicular phase	Unpublished
GSE6364	GPL570: [HG-U133_Plus_2]	54675	11 (05/06)	Follicular phase/Uterus-Proliferative	Burney et al., 2007
GSE51981	GPL570: [HG-U133_Plus_2]	54675	64 (35/29)	Follicular phase/Uterus-Proliferative	Tamaresis et al., 2014
GSE11691	GPL96: [HG-U133A]	22283	18 (09/09)	Follicular phase/Uterus-Proliferative	Hull et al., 2008
GSE7846	GPL570: [HG-U133_Plus_2]	54675	10 (05/05)	Follicular phase/Uterus-Proliferative	Sha et al., 2007
GSE25628	GPL571: [HG-U133A_2]	22277	22 (15/07)	Follicular phase/Uterus-Proliferative	Crispi et al., 2013
GSE6364	GPL570: [HG-U133_Plus_2]	54675	09 (03/06)	Lutal phase/Uterus-Secretory phase (Early)	Burney et al., 2007
GSE51981	GPL570: [HG-U133_Plus_2]	54675	30 (12/18)	Lutal phase/Uterus-Secretory phase (Early)	Tamaresis et al., 2014
GSE6364	GPL570: [HG-U133_Plus_2]	54675	17 (08/09)	Lutal phase/Uterus-Secretory phase (Mid)	Burney et al., 2007
GSE51981	GPL570: [HG-U133_Plus_2]	54675	50 (22/28)	Lutal phase/Uterus-Secretory phase (Mid)	Tamaresis et al., 2014
Total			292 (147/145)		
**(B) MULTIPLE SCLEROSIS (MS)**					
GSE38010	GPL570: [HG-U133_Plus_2]	33398	07 (02/05)	Brain/Early, active and late stage of MS	Han et al., 2012
GSE26484	GPL570: [HG-U133_Plus_2]	54675	10 (04/06)	Peripheral blood/Low & high serum sema4A levels	Nakatsuji et al., 2012
GSE21942	GPL570: [HG-U133_Plus_2]	54675	29 (15/14)	Peripheral blood/NA	Kemppinen et al., 2011
GSE41890	GPL6244: [HuGene-1_0-st]	695	36 (12/24)	Peripheral blood/Remission & Relapse	Irizar et al., 2014
GSE16461	GPL570: [HG-U133_Plus_2]	54675	16 (08/08)	Peripheral blood/Monozygotic twins (MZ)	Annibali et al., 2011
GSE41849	GPL16209: [HG Exon 1.0 ST]	18725	25 (13/12)	Whole blood/Baseline	Nickles et al., 2013
GSE41849	GPL16209: [HG Exon 1.0 ST]	18725	24 (12/12)	Whole blood/Follow-up year 1	Nickles et al., 2013
GSE41848	GPL16209: [HG Exon 1.0 ST]	18725	69 (28/41)	Whole blood/Baseline	Nickles et al., 2013
GSE41848	GPL16209: [HG Exon 1.0 ST]	18725	88 (31/57)	Whole blood/Follow-up year 1/2	Nickles et al., 2013
Total			304 (125/179)		

### Data preprocessing and mining of DEGs

We used GEO2R web tool (http://www.ncbi.nlm.nih.gov/geo/geo2r/; Barrett et al., 2013) to compare two or more groups of samples in a GEO profile (given in Table 1) in order to identify genes that are differentially expressed across the diverse experimental conditions (Wu et al., 2016; Mou et al., 2017; Sun et al., 2017). GEO2R performs comparisons on original submitter-supplied processed/normalized data tables using the GEOquery and limma (Linear Models for Microarray Analysis) R packages (Smyth, 2004). The adjusted *p*-values (adj. P) using Benjamini and Hochberg (1995) false discovery rate (FDR) method by default were used to correct for the occurrence of false positive results. The threshold value for identifying DEGs was set as FDR ≤ 0.05 and logFC ≥ 1.5. All gene probes across the microarray datasets were converted to a common Entrez ID using the “Database for Annotation, Visualization, and Integrated Discovery” (DAVID) v6.7 tool (https://david.ncifcrf.gov/conversion.jsp; Huang da et al., 2008, 2009). The probes not associated with known genes were not included. The differentially expressed genes (DEGs) from individual studies were selected using a combination of *p*-value and fold change and the results were combined by taking the union of all individual studies. When multiple probes referred to the same gene, the expression values obtained from these probes were minimized to a single value by averaging the expression value (when all genes with the same direction of expression) or discarded (when genes had diverse direction of expression). The resulting DEGs represent the entire gene set of all studies.

### Functional and pathway enrichment analysis of DEGs

KEGG and GO (Gene Ontology) analysis were completed separately using the DAVID tool. DAVID uses Fisher's exact test (Fisher, 1922) to predict enriched pathways by gene-set enrichment analysis of Kyoto Encyclopedia of Genes and Genomes (KEGG) database. False Discovery Rate (FDR) adjusted *p* ≤ 0.05 was selected for significantly over-represented pathways. Significant pathway results were ranked according to the *p*-value. In this study, GO terms from the biological process ontology were analyzed. The GO terms with ≥5 numbers of genes and adjusted *p* ≤ 0.05 were considered significantly enriched GO terms. The shared biological process ontology (GO-BP) was calculated based on overlapping GO-Ids between EMS and MS. The number of genes enclosed by sharing GO terms was used to predict the significant pathways and ensuing crosstalk between these pathways. Cytoscape plugin BiNGO (Maere et al., 2005) and FunRich V3 (Pathan et al., 2015) stand alone software was separately used for functional enrichment analysis of DEGs.

### PPI network-based enrichment analysis

To expose the interactive associations among the DEGs at the protein level, genes obtained from the EMS and MS were mapped on protein-protein interaction (PPI) data using NetworkAnalyst tool (http://www.networkanalyst.ca; Xia et al., 2015). The network building was limited to include only the original seed proteins by picking the zero order interactions to evade “hairball effect.” NetworkAnalyst integrates comprehensive PPI data from published literature with experimental information available across different PPI databases. These databases like IntAct (Orchard et al., 2014), MINT (Licata et al., 2012), DIP (Salwinski et al., 2004), BIND (Isserlin et al., 2011), and BioGRID (Chatr-Aryamontri et al., 2015) are integrated in InnateDB (Breuer et al., 2013). Topological properties (such as betweenness centrality and degree distribution) of the constructed PPI network were calculated by NetworkAnalyzer in Cytoscape (Shannon et al., 2003). The degree distribution of all nodes in the network may help to explain whether a network is scale-free or not. The betweenness centrality is defined as the number of shortest paths in the graph that pass through the node divided by the total number of shortest paths. The nodes with a high betweenness centrality are lying on the communication paths and can control the information flow. The densely connected group of proteins referred as modules in a given network was predicted using the “module explorer” panel of NetworkAnalyst that used a random walk based approach for module detection. The significant *p*-value of a given module was calculated using Wilcoxon rank-sum test (Haynes, 2013). The ranking of the identified modules was based on the number of encompassed seed proteins. The enriched pathways of DEGs in significant modules (≥10 DEGs) were analyzed with a threshold of *p* ≤ 0.05 using DAVID.

### Transcription factor and protein kinase associated with DEGs

Upstream regulators and protein kinases associated with DEGs were recognized by submitting the list of shared DEGs to Expression2Kinases (X2K) web interface (https://amp.pharm.mssm.edu/X2K/; Chen et al., 2012). X2K identifies the enriched transcription factors (TFs) from the upstream of the shared DEGs using a ChEA database (Chen et al., 2012). Genes2Networks (G2N) module of X2K connects TFs with PPI to yield transcriptional complexes related to these gene signatures. Protein Kinases responsible for TF complex formation and functional regulation were recognized through the Kinase Enrichment Analysis (KEA) module of X2K. Top 10 most enriched TFs and kinases were ranked based on the combined (*p*-value and z-score) score.

### Crosstalk analysis of biological pathways

Pathway crosstalk was defined as those pathways that had overlapping genes and edges. In this study, two pathways were considered to crosstalk if they comprised at least 5 DEGs with adjusted *p* ≤ 0.05 and at least 1 overlapping gene and edge. If the number of overlapped DEGs in the pathways was more than 3, the two pathways were considered to have a strong interaction. This criterion ensures that each of the pathways and its crosstalk pairs were statistically significant and contained biologically meaningful number of genes. The crosstalk between two pathways was explored by calculating the Jaccard Coefficient (JC)/Jaccard Index (JI) (Jaccard, 1912; Levandowsky and Winter, 1971) and Overlap Coefficient (OC)/Szymkiewicz-Simpson coefficient values (Vijaymeena and Kavitha, 2016). The pairs of the pathway were subsequently ranked by taking the average of the two measurements as the score defining pathway crosstalk.

The JC/JI or J (A, B) is represented by the formula

$$\text{J}\left(\text{A},\text{B}\right){=}^{\;}\frac{\left|\text{A}\cap \text{B}\right|}{\left|\text{A}\cup \text{B}\right|}=\frac{\left|\text{A}\cap \text{B}\right|}{\left|\text{A}\right|+\left|\text{B}\right|-|\text{A}\cap \text{B}|}$$

Here, in two given pathways, A and B, the overlapping number of genes is the intersection of path A and path B, and the union of the two paths represents the sum of genes. Pathways with JC ≥ 0.01 and sharing at least one DEG were considered.

The Overlap Coefficient (OC) is represented by the formula

$$\text{Overlap}\left(\text{A},\text{B}\right){=}^{\;}\frac{\left|\text{A}\cap \text{B}\right|}{\min\;(\left|\text{A}\right|,\left|\text{B}\right|)}$$

OC was used to determine the fraction of genes that were overlapped across pathways. The larger the OC, the higher is the similarity in gene information between two pathways. The degree of overlap was defined based on OC-values. OC = 1 represented a complete overlap, OC ≫50% indicated high overlap, 20% ≪ OC <50% moderate overlap, OC < 20% low overlap and OC = 0 was taken as no overlap among the two pathways.

## Results

### Identifying disease-associated DEGs from EMS and MS

**Figure 1:**
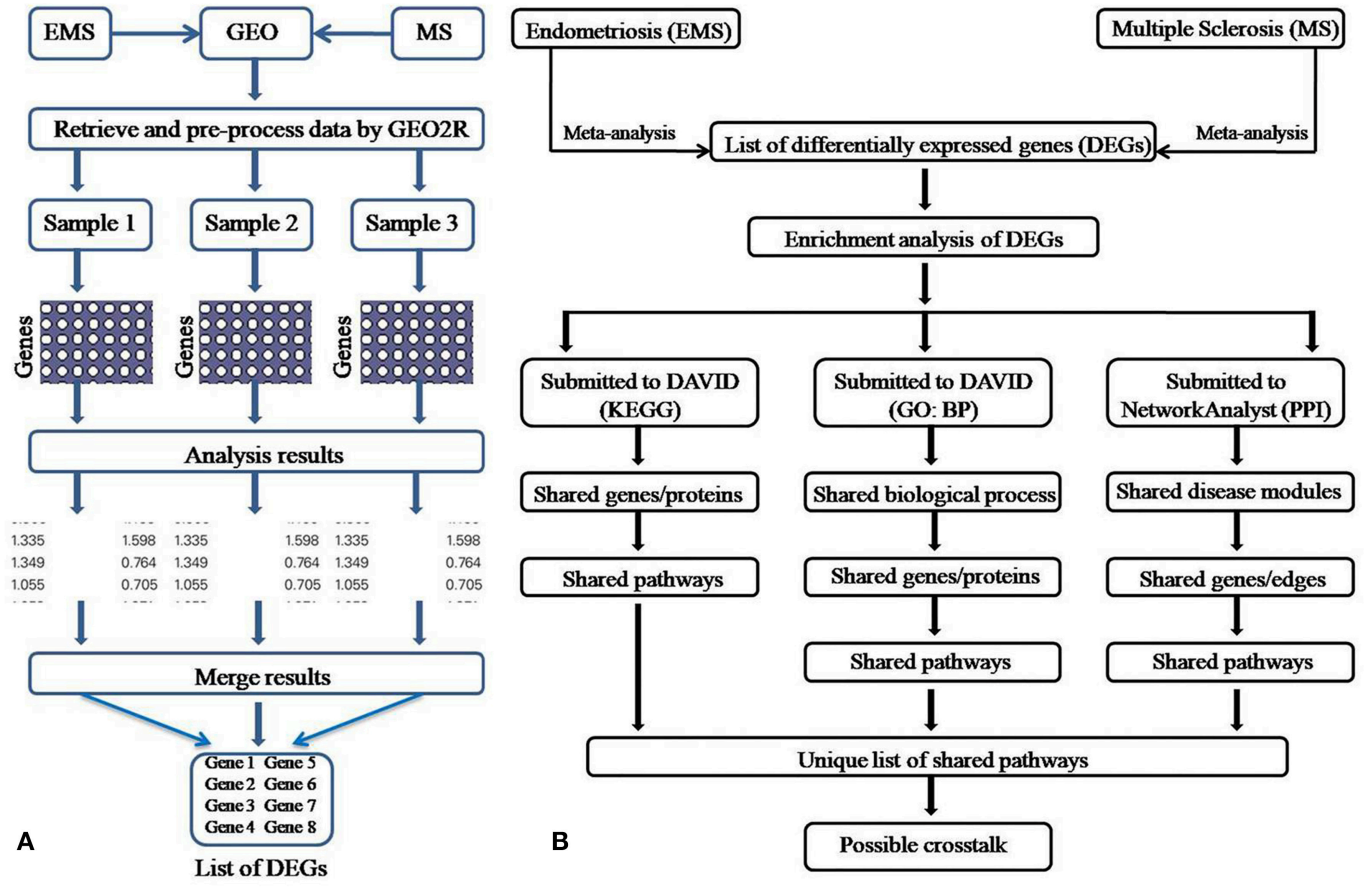
Pictorial depiction of the meta-analysis **(A)** and enrichment analysis **(B)** method adopted in this study.

The meta-analysis approach (Arasappan et al., 2011; Taminau et al., 2014) for the integrative analysis of multiple gene expression profiles (Figure 1A) was adopted in this study. Primary screening of the GEO profiles offered 292 and 304 samples in EMS and MS respectively, irrespective of the location of tissue sampling (Table 1). R/Bioconductor limma package (Smyth, 2004) was used to predict the DEGs between disease and control samples in each dataset and the identified DEGs were merged by taking the union of all individual studies. The genes which are expressed in these studies in the same direction for both diseases were averaged and retained, whereas genes which were observed in opposite directions were discarded. The final dataset represents the entire gene set of all studies (Table S1). The probe annotated as antisens RNA, miRNA, chromosomes, hypothetical, loci, non-coding RNAs, non-functional proteins, non-protein coding genes, ORF, pseudogenes, and uncharacterized genes were not considered. As a result, a total of 711 (corresponding to 796 probes) and 1,516 (corresponding to 1,833 probes) DEGs were obtained in EMS and MS patients, respectively (Table S2). The candidate genes were ranked according to their differential expression in response to disease samples as compared to control samples (Table 2). Out of the 711 EMS-associated genes, 254 genes were up-regulated and 457 genes were down-regulated. In contrast, 779 genes were up-regulated and 737 genes were down-regulated in MS. The common genes shared by both EMS and MS were observed to be 129 genes (Figure 2A). The greatest fold differential expression observed was a 4.67 fold up-regulation of the RBMS3 gene (binding motif, single stranded interacting protein 3) and a 4.68 fold down-regulation of the SCD gene (stearoyl-CoA desaturase/delta-9-desaturase) in MS as compared with EMS. The acquired DEGs from this study were mapped to the validated disease genes of endometriosis and MS offered in Online Mendelian Inheritance in Man (OMIM) (Amberger et al., 2014) and DisGeNET (Piñero et al., 2015) human genetic disorder databases. This analysis confirmed that 58 and 109 validated genes (Figure 2B, Tables S3, S4) present in these two databases had also been identified as EMS and MS-linked DEGs, respectively in our datasets. This revealed that the identified DEGs were appropriate to signify the two disease conditions.

**Table 2:** Top 10 differentially expressed genes identified in meta-analysis.

^*^Entrez ID	**Gene symbol**	**Gene name**	**Fold change (FC)**		**^#^Adjusted *p*-value**	
**(A) Endometriosis (EMS)**						
**TOP 10 UP-REGULATED GENES**						
170302	ARX	Aristaless related homeobox	6.16		7.60E-16	
**5010**	CLDN11	Claudin 11	6.08		2.53E-13	
55026	TMEM255A	Transmembrane protein 255A	4.66		8.09E-10	
1295	COL8A1	Collagen type VIII alpha 1 chain	4.49		2.25E-08	
6332	SCN7A	Sodium voltage-gated channel alpha subunit 7	3.92		2.23E-06	
**4915**	NTRK2	Neurotrophic receptor tyrosine kinase 2	3.54		5.72E-07	
345557	PLCXD3	Phosphatidylinositol specific phospholipase C X domain containing 3	3.41		7.69E-09	
23224	SYNE2	Spectrin repeat containing nuclear envelope protein 2	3.39		1.63E-10	
55220	KLHDC8A	Kelch domain containing 8A	3.35		1.09E-02	
5549	PRELP	Proline and arginine rich end leucine rich repeat protein	3.35		1.43E-04	
**TOP 10 DOWN-REGULATED GENES**						
115111	SLC26A7	Solute carrier family 26 member	−4.29		2.28E-08	
56547	MMP26	Matrix metallopeptidase 26	−4.25		3.34E-04	
9848	MFAP3L	Microfibril associated protein 3	−3.75		1.81E-03	
**100133941**	CD24	CD24 molecule	−3.42		2.01E-06	
57535	KIAA1324	KIAA1324	−3.42		3.38E-05	
3170	FOXA2	Forkhead box A2	−3.29		1.37E-07	
1750	DLX6	Distal-less homeobox 6	−3.20		7.93E-05	
3213	HOXB3	Homeobox B3	−3.19		1.16E-08	
64321	SOX17	SRY-box 17	−3.15		1.19E-05	
27324	TOX3	TOX high mobility group box family member 3	−3.10		2.20E-02	
**(B) Multiple sclerosis (MS)**						
**TOP 10 UP-REGULATED GENES**						
3706	ITPKA	Inositol-trisphosphate 3-kinase A	6.63		2.24E-02	
57495	NWD2	NACHT and WD repeat domain containing 2	6.28		2.24E-02	
9312	KCNB2	Potassium channel, voltage gated Shab related subfamily B, member 2	6.05		2.55E-02	
9495	AKAP5	A kinase (PRKA) anchor protein 5	5.94		3.16E-02	
**59350**	RXFP1	Relaxin/insulin-like family peptide receptor 1	5.86		1.63E-02	
2845	GPR22	G protein-coupled receptor 22	5.75		5.03E-02	
29953	TRHDE	Thyrotropin-releasing hormone degrading enzyme	5.34		2.35E-02	
**118427**	OLFM3	Olfactomedin 3	5.10		3.26E-02	
440279	UNC13C	unc-13 homolog C (*C. elegans*)	5.08		2.82E-02	
2561	GABRB2	Gamma-aminobutyric acid (GABA) A receptor, beta 2	5.05		2.46E-02	
**TOP 10 DOWN-REGULATED GENES**						
116835	HSPA12B	Heat shock 70kD protein 12B	−15.90		5.19E-02	
7276	TTR	Transthyretin	−8.19		3.97E-02	
1586	CYP17A1	Cytochrome P450, family 17, subfamily A, polypeptide 1	−6.75		2.12E-02	
**3284**	HSD3B2	Hydroxy-delta-5-steroid dehydrogenase, 3 beta- and steroid delta-isomerase 2	−6.58		1.36E-02	
8788	DLK1	Delta-like 1 homolog (Drosophila)	−6.13		2.12E-02	
53637	S1PR5	Sphingosine-1-phosphate receptor 5	−5.92		7.78E-03	
8513	LIPF	Lipase F, gastric type	−5.57		2.24E-02	
**3126**	HLA-DRB4	Major histocompatibility complex, class II, DR beta 4	−5.55		3.08E-02	
5015	OTX2	Orthodenticle homeobox 2	−5.30		2.06E-02	
1584	CYP11B1	Cytochrome P450, family 11, subfamily B, polypeptide 1	−5.18		2.61E-02	
**(C) Shared DEGs between EMS and MS**						
^*^Entrez ID	**Gene symbol**	**Gene name**	**Fold change (FC)**		**^#^Adjusted *p*-value**	
			**EMS**	**MS**	**EMS**	**MS**
**TOP 10 UP-REGULATED GENES**						
343450	KCNT2	Potassium channel, sodium activated subfamily T, member 2	2.71	4.08	4.15E-08	3.32E-02
1301	COL11A1	Collagen, type XI, alpha 1	2.60	4.18	3.49E-05	2.12E-02
257194	NEGR1	Neuronal growth regulator 1	2.19	4.32	1.81E-06	4.02E-02
27303	RBMS3	RNA binding motif, single stranded interacting protein 3	1.72	4.67	3.20E-03	2.06E-02
4057	LTF	Lactotransferrin	2.39	3.94	3.18E-04	1.74E-07
345557	PLCXD3	Phosphatidylinositol-specific phospholipase C, X domain containing 3	3.41	2.85	7.69E-09	3.51E-02
627	BDNF	Brain-derived neurotrophic factor	2.67	3.50	7.37E-08	4.29E-02
1131	CHRM3	Cholinergic receptor, muscarinic 3	1.65	4.37	3.33E-03	2.95E-02
283078	MKX	Mohawk homeobox	3.16	2.82	2.95E-07	2.12E-02
2823	GPM6A	Glycoprotein M6A	2.10	3.73	2.11E-04	2.76E-02
**TOP 10 DOWN-REGULATED GENES**						
7368	UGT8	UDP glycosyltransferase 8	−2.81	−4.30	2.11E-06	5.02E-02
6319	SCD	WSC domain containing 2	−1.51	−4.68	6.18E-04	3.59E-02
2065	ERBB3	Erb-b2 receptor tyrosine kinase 3	−2.56	−3.41	3.99E-05	2.11E-02
135932	TMEM139	Transmembrane protein 139	−1.72	−3.71	4.39E-03	4.57E-02
57475	PLEKHH1	Pleckstrin homology domain containing, family H (with MyTH4 domain) member 1	−1.85	−3.57	6.15E-03	2.79E-02
6299	SALL1	Spalt-like transcription factor 1	−2.35	−2.61	3.81E-10	4.12E-02
3092	HIP1	Huntingtin interacting protein 1	−2.14	−2.73	6.53E-04	2.92E-02
4678	NASP	Nuclear autoantigenic sperm protein (histone-binding)	−2.35	−2.08	1.18E-03	3.68E-02
9043	SPAG9	Sperm associated antigen 9	−1.53	−2.85	2.98E-03	3.08E-02
3696	ITGB8	Integrin, beta 8	−1.61	−2.73	1.73E-03	5.25E-02

* *Bold and underlined gene IDs denoted to the existence of these genes in OMIM and DisGeNET disease databases*.

# *p-values are adjusted, based on the False Discovery Rate (FDR) using the Benjamini–Hochberg method*.

**Figure 2:**
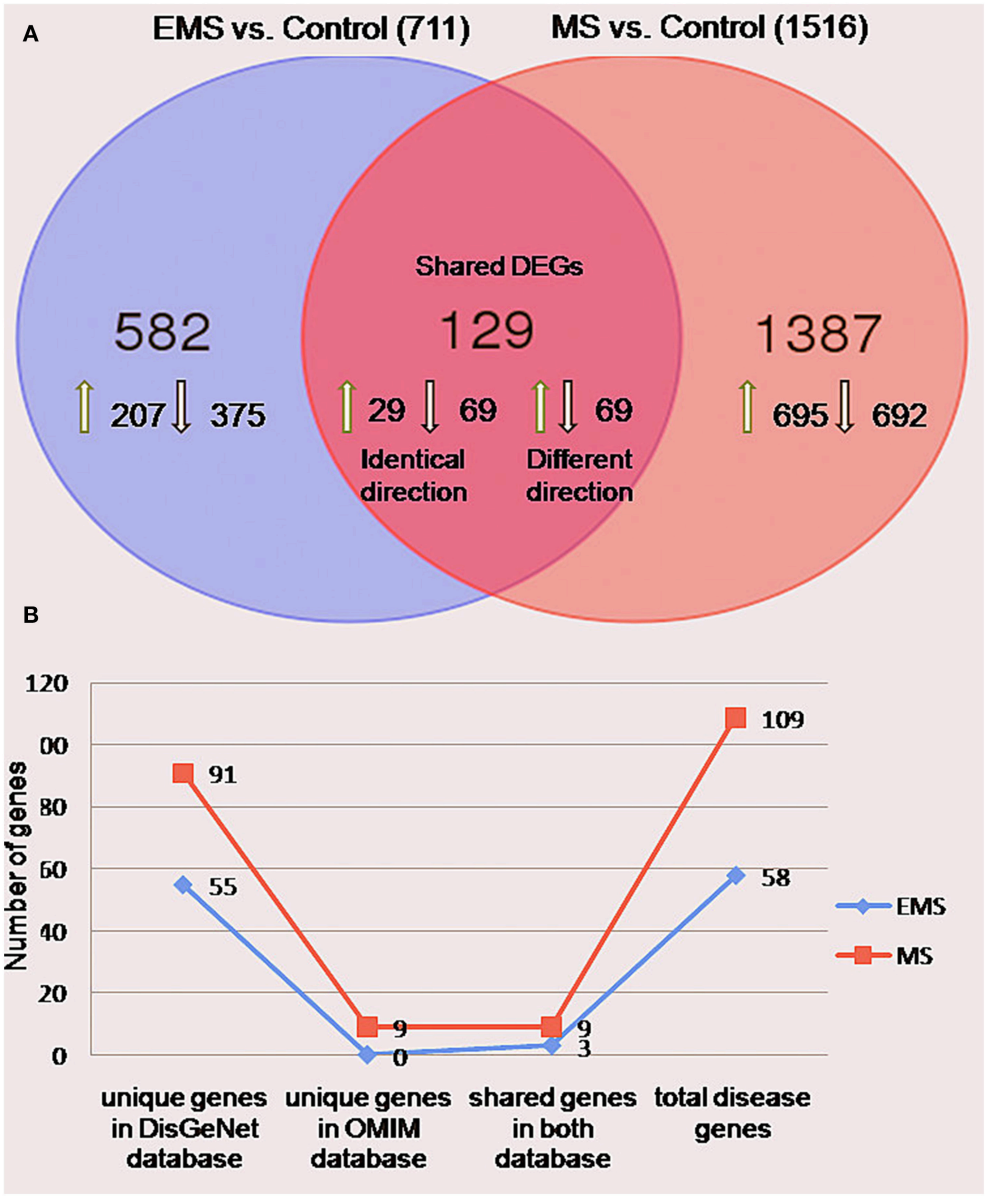
Differentially expressed genes in the group of endometriosis vs. control and multiple sclerosis vs. control. **(A)** Venn diagram indicating the number of uniquely upregulated (green arrow) or downregulated (black arrow) genes compared individuals with EMS and MS to normal persons. The findings revealed 711 (32%) and 1516 (68%) genes in EMS and MS, respectively with 129 shared DEGs between them. **(B)** A line chart showed the occurrence of 58 (3%) and 109 (5%) genes in OMIM and DisGeNET disease databases.

### Identifying over-represented pathways and biological process

**Figure 3:**
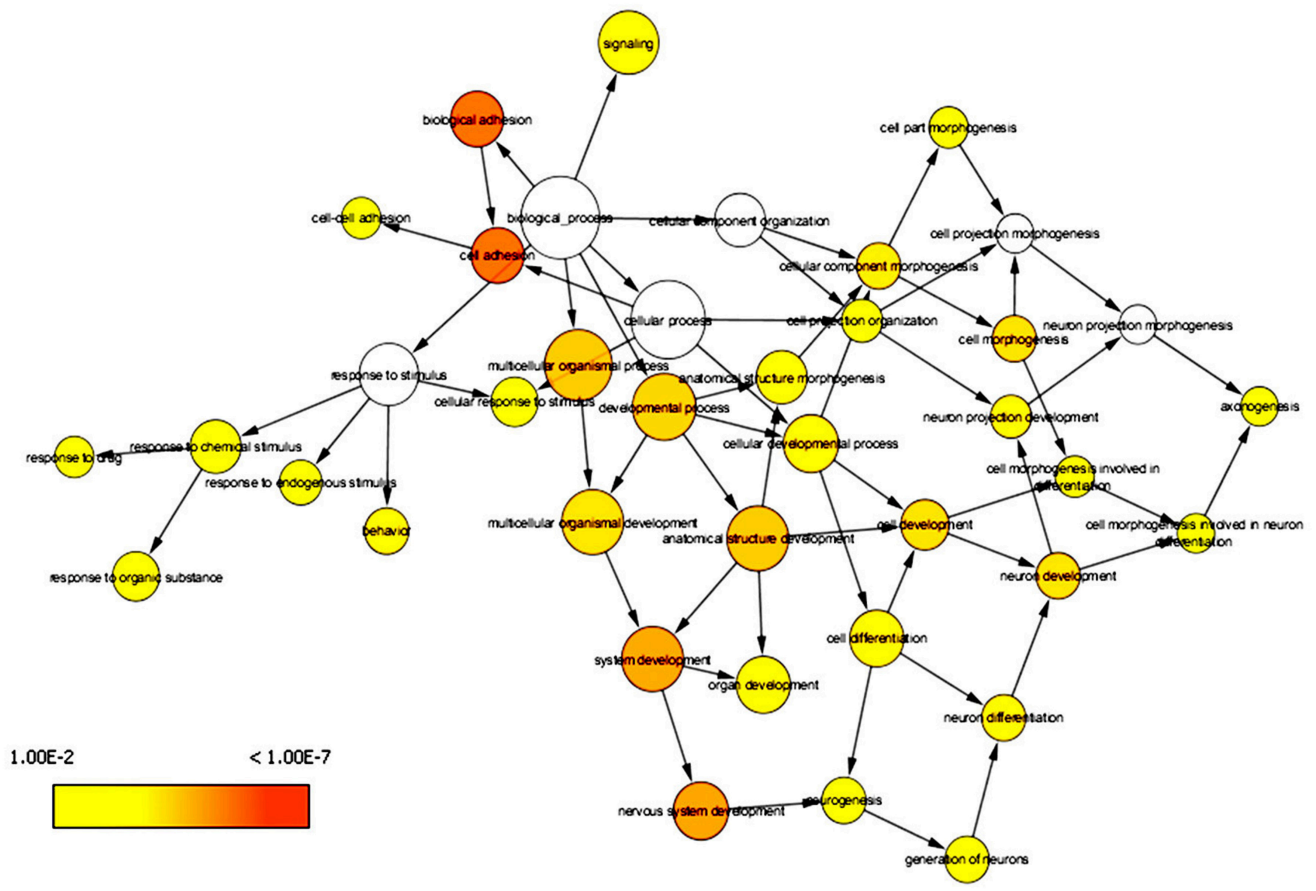
Enrichment network of shared DEGs based on biological processes. Significantly overrepresented biological processes ontology terms were analyzed and visualized by Cytoscape plugin BiNGO. The structure of GO is described in terms of a graph, where each GO term is a node, and the relationships between the terms are edges indicating parent-to-child relationships. The size of a node is proportional to the number of targets in the GO category. The color denotes enrichment significance-the deeper the color on a color scale, the higher the enrichment significance.

**Figure 4:**
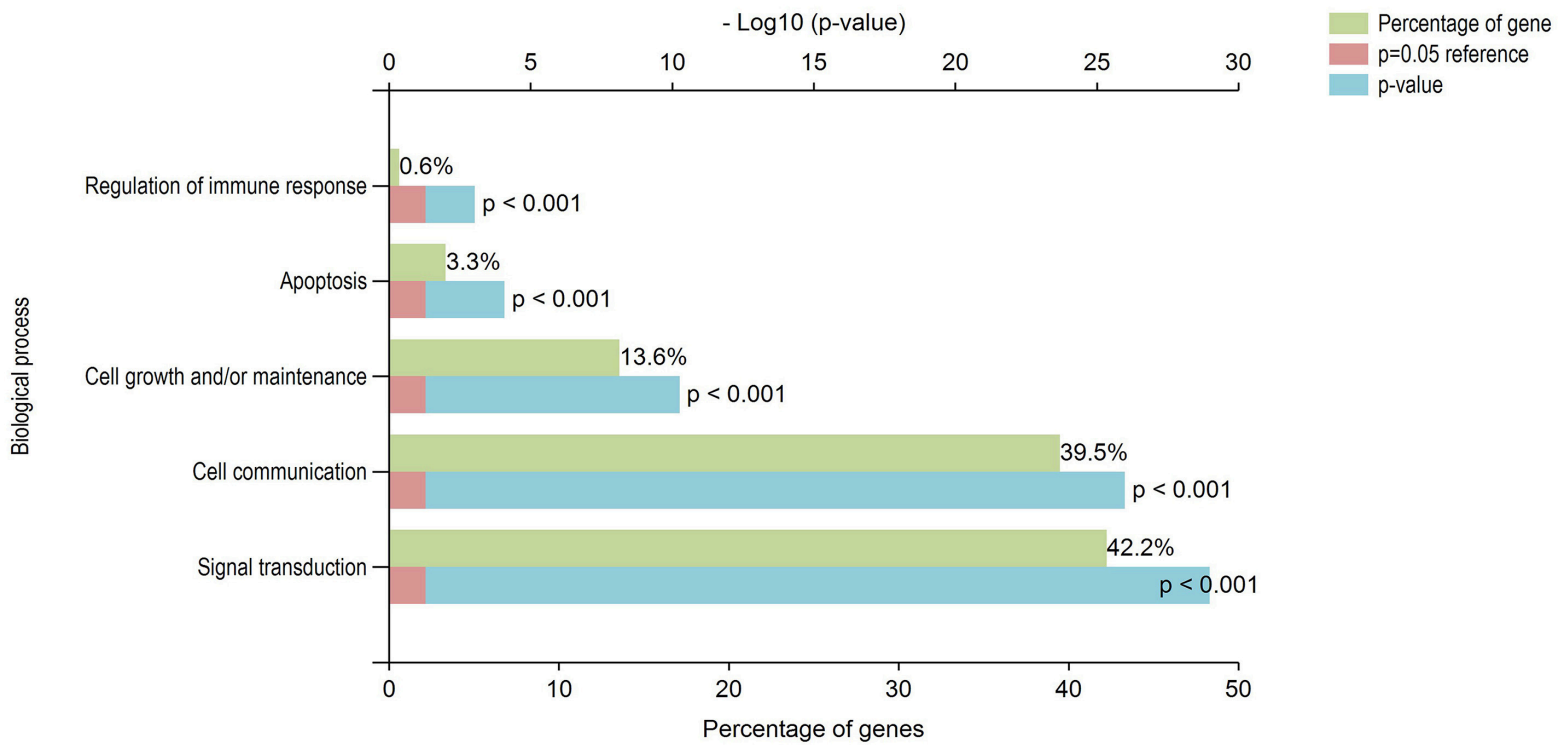
Gene ontology functional analyses of DEGs. Biological significance of DEGs was documented by enriching the GO terms of biological processes. Majority of the identified DEGs, from both up-regulated and down-regulated genes were involved in inflammatory/immune responses related functions. The green, blue and red colors represent the percentage of genes, *p*-value and reference (*p* = 0.05), respectively.

DAVID has the capability to independently execute Gene ontology (GO) and pathway enrichment analyses. Hence, GO and pathway (KEGG) enrichment analyses of identified DEGs were separately performed using DAVID. Initially, a complete set of up-regulated and down-regulated genes from EMS and MS were mapped to the terms in the KEGG database (Kanehisa et al., 2016, 2017). The statistical cut-off criterion of *p* ≤ 0.05 acknowledged 17 over-represented pathways commonly altered in both EMS and MS. These dysregulated pathways were found to be enriched with 26 overlapping genes in both diseases (Figure S1 and Table S5). The top five enriched pathways based on shared genes were cell adhesion molecules (hsa04514, 7 DEGs), calcium signaling pathway (hsa04020, 7 DEGs), focal adhesion (hsa04510, 4 DEGs), tight junction (hsa04530, 4 DEGs) and dilated cardiomyopathy (hsa05414, 3 DEGs). The shared pathways enriched with most of the DEGs were associated with inflammatory/immune responses. Similarly, biological processes common to both diseases were identified by mapping up-regulated and down-regulated genes to various GO categories in the GO database. To minimize false-positives, a GO biological process was considered significantly enriched if it contained a minimum number of five genes with *p* < 0.05. The DEGs from EMS and MS were clustered into 136 and 215 functional groups, respectively (Tables S6A,B). Twenty eight over-represented GO terms of biological processes were found to be affected commonly in both EMS and MS (Table S6C). Cytoscape plugin BiNGO was used to represent significantly overrepresented GO terms in an enrichment network (Figure 3). The top five shared biological process GO terms were cell adhesion (GO:0007155, 17 DEGs), biological adhesion (GO:0022610, 16 DEGs), neuron differentiation (GO:0030182, 11 DEGs), regulation of apoptosis (GO:0042981, 11 DEGs), and cell morphogenesis (GO:0000902, 10 DEGs). To further explore the biological process of the shared DEGs, a separate enrichment analysis using stand-alone FunRich V3 software was completed. As a result, signal transduction (42.2% DEGs), cell communication (39.5% DEGs), cell growth and/or maintenance (13.6% DEGs), apoptosis (3.3% DEGs) and regulation of immune response (0.6% DEGs) related GO terms were observed to be significantly overrepresented in both diseases (Figure 4). These results also revealed that most of the shared biological processes enriched with maximum number of genes were related to inflammation/immune response. Genes associated with enriched GO terms were then mapped onto the corresponding KEGG pathway. The analysis yielded 30 significant pathways associated with biological processes (386 GO terms) of commonly altered DEGs in both diseases (Table S7A, Figure S2). The top five enriched pathways based on shared DEGs of biological processes were focal adhesion (hsa04510, 19 DEGs), pathways in cancer (hsa5200, 18 DEGs), ECM-receptor interaction (hsa04512, 17 DEGs), ErbB signaling pathway (hsa04012, 16 DEGs), and neurotrophin signaling pathway (hsa04722, 16 DEGs). The dysregulated pathways mainly affected by shared biological processes were associated with the inflammatory/immune responses.

### Identifying hub genes in protein interaction networks

Protein–protein interactions (PPI) network was constructed by mapping abnormally expressed genes from EMS and MS with PPI data to predict biological significant modules comprising shared genes/proteins and interactions which were likely to play key roles in linking EMS and MS. A zero-order interaction network comprising seed nodes only with interconnected edges was constructed to generated interactions for integrated DEGs. The resulting PPI network scattered in 1–20 subnetwork including one large network with highest nodes and edges. We investigated the network features of large PPI subnetwork using NetworkAnalyst and Cytoscape plug-in “NetworkAnalyzer” that accumulated as an undirected graph (edges have no direction), where the proteins/genes were denoted with “nodes” and the interaction between any two proteins/genes was denoted by “edge.” The results disclosed the associations of 1,029 nodes (49.05% of DEGs) and 2136 edges in the network (Figure S3). We calculated the degree of the genes in the PPI network and found 372 (36.15%) with a degree of one and 657 (63.85%) with a degree greater than one. Out of 657 nodes, a total of 85 nodes were observed with ≥10 connections with other nodes. We observed “betweenness” with a range of 2.5 to 212999.72 for large number of nodes (626; 60.84%) in the constructed network. These observations suggested that hub (high degree or number of connections it has to other nodes) and bottleneck (high betweenness or number of shortest paths going through the node) proteins which are likely to be essential proteins were abundant in the constructed network (Yu et al., 2007; Raman, 2010). The genes, APP (degree 207; betweenness centrality 212999.72), HSP90AB1 (degree 70; betweenness centrality 52964.54), CTNNB1 (degree 57; betweenness centrality 55223.07), and MDM2 (degree 51; betweenness centrality 45486.22) among the down-expressed DEGs, whereas SUMO1 (degree 87; betweenness centrality 75089.82) and EGR1 (degree 70; betweenness centrality 45796.81) among the over-expressed DEGs were observed to be the most highly ranked hub genes in this study. The results suggest that proteins with the highest degree in the network have the highest betweenness (Table S8A). As hubs contributed in a number of interactions and clutch the network together (Jeong et al., 2001), they are more likely to be master regulators of signaling and transcription. Therefore, the hubs can prove to be helpful as therapeutic targets or biomarkers.

### Identifying disease module through interaction networks

**Figure 5:**
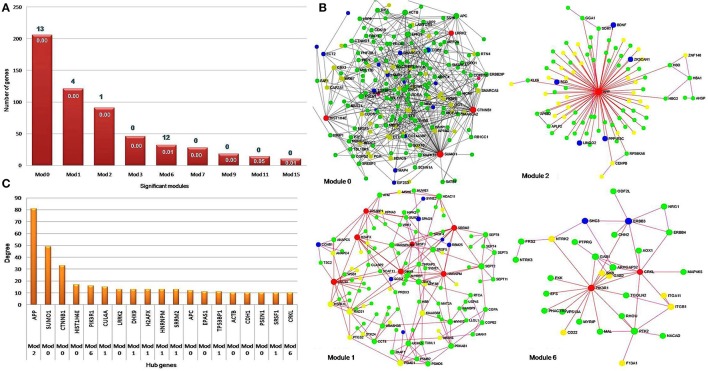
Hub genes in significant network modules. **(A)** The column chart shows the size of significant modules based on the number of genes they contain. Four modules, namely zero, one, two and six were found to be enriched with 25 pathways. **(B)** The network modules show a high degree of clustering of proteins involved in the identical disease. The hub genes with high degree and high betweenness were denoted with red color. The EMS, MS, and shared DEGs in the PPI network modules were denoted by the color yellow, green and blue, respectively. **(C)** The bar diagram represents the distribution of hub genes with high degree i.e., hub genes ≥ 10 nodes. The hub gene APP (amyloid beta precursor protein) was found to be connected with the highest number of neighbor's node i.e., 81 in the network.

**Figure 6:**
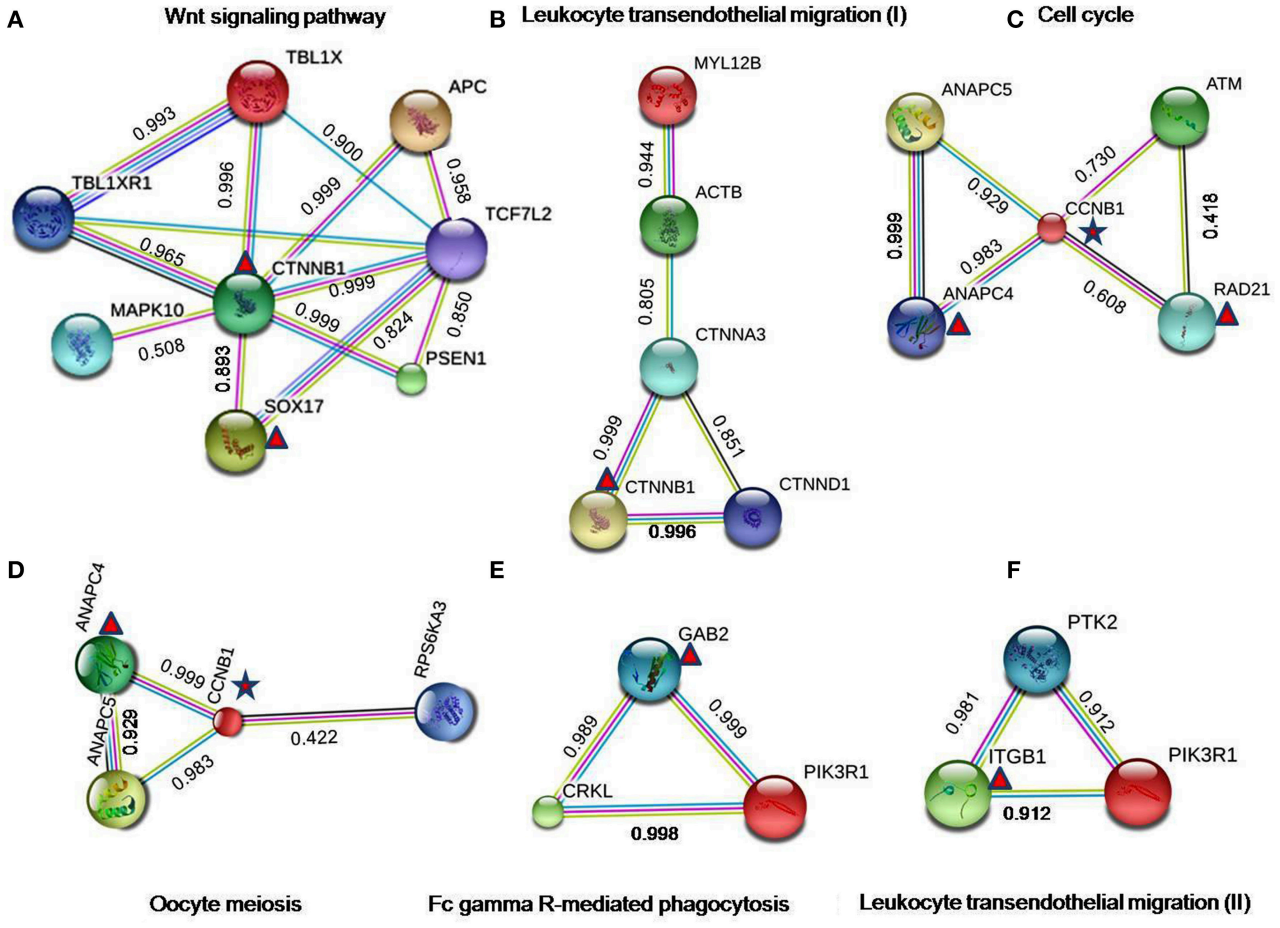
Protein-protein interactions in crosstalk pathways. The results of the PPI analysis disclosed a number of interactions/crosstalk through overlapping edges in EMS and MS (shown in the figure from **(A–F)**. The triangular symbol denoted to the DEGs from endometriosis, whereas star symbol indicated to the shared DEGs between EMS and MS. The interactions score indicates the interaction confidence between two nodes. All scores rank from 0 to 1, with 1 being the highest possible confidence. The color of edges in the PPI network correspond to the curated databases (blue), and experimentally determined (pink) from known interactions, while gene neighborhood (green), gene fusions (red), and gene co-occurrence (blue) signify predicted interactions. Further representations include text mining (yellow), co-expression (black), and protein homology (light blue).

The constructed PPI network was evaluated for module detection, which contains a group of proteins that execute similar functions. Thirty four highly connected independent modules were observed. We observed nine overlapping modules with more than 10 nodes (*p* ≤ 0.05) (Table S8B), signifying possible interaction, shared by the two diseases (Samanta and Liang, 2003; Menche et al., 2015; Caldera et al., 2017). The predicted modules were connected to neighboring modules and ranged in size from 10 to 206 genes. The distribution of highly connected hub nodes (degree ≥ 10) in modules gave highest hub nodes of 9 genes (SUMO1, CTNNB1, HIST1H4E, LRRK2, APC, EPAS1, ACTB, CDH1, and PSEN1) in module 0. The next hub nodes of 7 genes (CUL4A, HNRNPM, DHX9, SRRM2, H2AFX, TP53BP1, and SRSF1) occurred in module 1. An evaluation of the modules by KEGG database exposed a number of dysregulated pathways with an FDR ≤ 0.05. Modules 0, 1, 2, and 6 emerged with shared interactions through 25 commonly dysregulated pathways (Figures 5A–C, Table 3, Table S9). An examination at the pathway level of PPI connecting EMS and MS yielded significant crosstalk through nine overlapping genes and 44 overlapping edges (Figures 6A–F). We also found several genes/proteins were involved in multiple pathways indicating that these PPIs might link the crosstalk pathways together.

**Table 3:** Crosstalk interactions through overlapping edges in pathways that is significantly dysregulated (*P* < 0.05) in both EMS and MS.

**KEGG ID**	**Pathway**	**Classification**	**Hits**	**Node**	**Edge**	**DEGs in PPI network**	**Figure**
hsa04310	Wnt signaling pathway	Signal transduction	8	8	13	^&^TBL1XR1; ^&^TBL1X; ^&^APC; ^&^TCF7L2; ^&^PSEN1; ^&^MAPK10 ; ^$^SOX17; ^$^CTNNB1	Figure 6A
hsa04670	Leukocyte transendothelial migration (a)	Immune system	5	5	2	^&^ACTB; ^&^CTNND1; ^&^CTNNA3; ^&^MYL12B ; ^$^CTNNB1	Figure 6B
hsa04110	Cell cycle	Cell growth and death	5	5	6	^&^ATM; ^&^ANAPC5 ; ^$^RAD21; ^$^ANAPC4; ^#^CCNB1	Figure 6C
hsa04114	Oocyte meiosis	Cell growth and death	4	4	4	^&^RPS6KA3; ^&^ANAPC5; ^$^ANAPC4; ^#^CCNB1	Figure 6D
hsa04666	Fc gamma R-mediated phagocytosis	Immune system	3	3	3	^&^CRKL; ^&^PIK3R1 ; ^$^GAB2	Figure 6E
hsa04670	Leukocyte transendothelial migration (b)	Immune system	3	3	3	^&^PTK2; ^&^PIK3R1; ^$^ITGB1	Figure 6F

$ Genes from EMS;

& genes from MS;

# *shared genes between EMS and MS*.

*ACTB, actin, beta; ANAPC4, anaphase promoting complex subunit 4; ANAPC5, anaphase promoting complex subunit 5; APC, adenomatous polyposis coli; ATM, ataxia telangiectasia mutated; CCNB1, cyclin B1; CRKL, v-crk sarcoma virus CT10 oncogene homolog (avian)-like; CTNNA3, catenin (cadherin-associated protein), alpha 3; CTNNB1, catenin (cadherin-associated protein), beta 1, 88kDa; CTNND1, catenin (cadherin-associated protein), delta 1; GAB2, GRB2-associated binding protein 2; ITGB1, integrin, beta 1; MAPK10, mitogen-activated protein kinase 10; MYL12B, myosin, light chain 12B; PIK3R1, phosphoinositide-3-kinase, regulatory subunit 1 (alpha); PSEN1, presenilin 1; PTK2, PTK2 protein tyrosine kinase 2; RAD21, RAD21 homolog (S. pombe); RPS6KA3, ribosomal protein S6 kinase, 90kDa, polypeptide 3; SOX17, SRY (sex determining region Y)-box 17; TBL1X, transducin (beta)-like 1X-linked; TBL1XR1, transducin (beta)-like 1 X-linked receptor 1; TCF7L2, transcription factor 7-like 2 (T-cell specific, HMG-box)*.

### Upstream regulator analysis of up- and down-regulated DEGs

**Figure 7:**
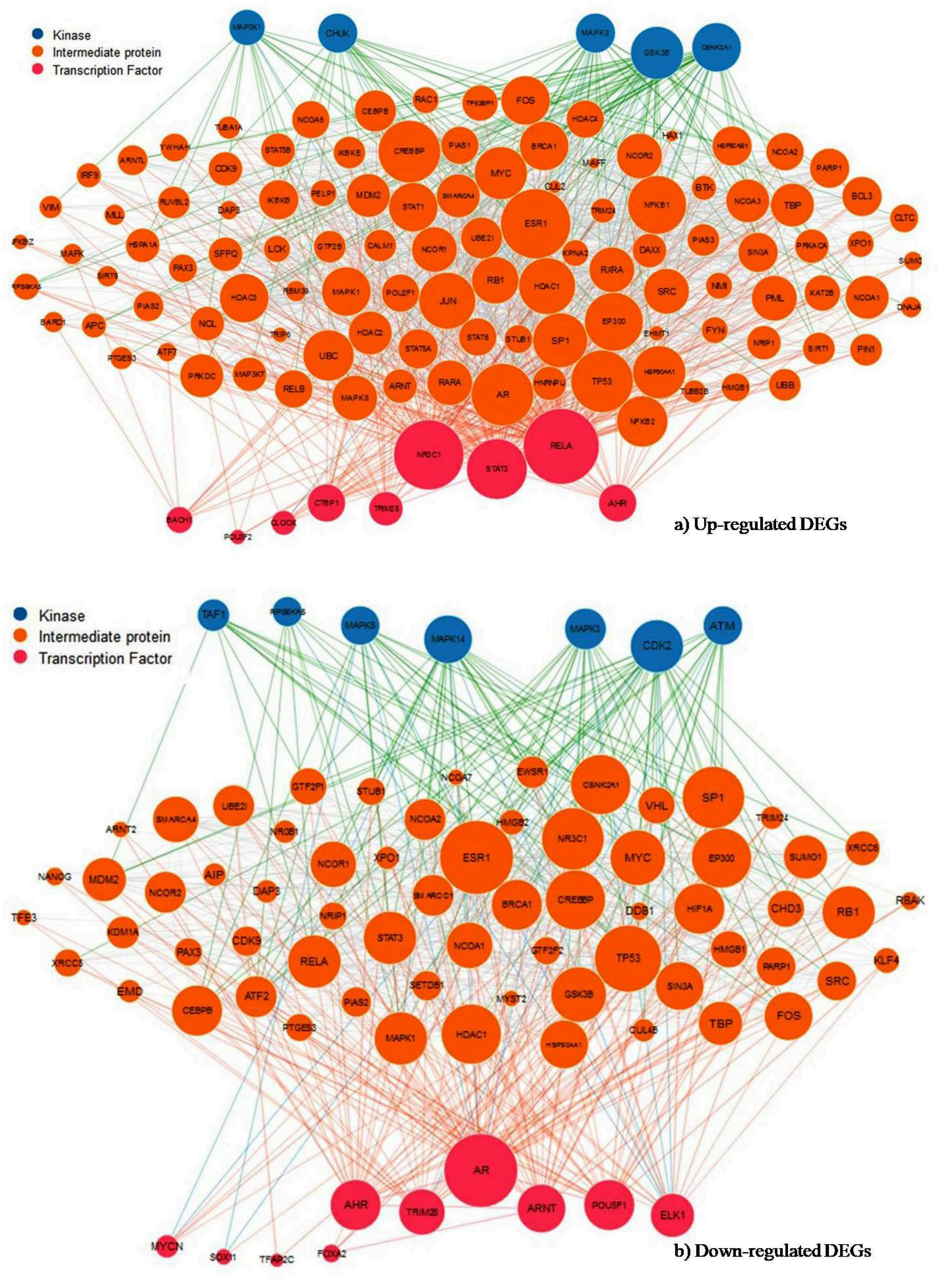
Subnetwork of transcription factor, intermediate protein and protein kinase. Expression2Kinases analysis of the **(A)** up- and **(B)** down-regulated genes signify the most enriched TFs and kinase from the upstream of shared DEGs based on the combined score (*p*-value and *z*-score). Node size reflects connectivity and color distinguishes transcription factors in pink, intermediate proteins in orange and kinases in blue.

The current study identifies the enriched upstream transcription factor (TF), intermediate protein and associated protein kinase to understand the mechanisms of shared up-regulated and down-regulated genes in both EMS and MS. The X2K approach revealed that POU3F2, BACH1 and 3 were top TFs binding to up-regulated genes (Figure 7A), whereas SOX11, AR and TRIM28 were the leading TFs in down-regulated genes (Figure 7B, Table 4). The comparison of TFs of both upregulated and down-regulated genes resulted in the overall divergence of TFs except for AHR and TRIM28. These TFs were found to be connected with 411 and 199 intermediate proteins for up-regulated and down-regulated genes, respectively. These intermediate proteins might be facilitating the TFs to be active in the cells. The study also disclosed the enriched protein kinases such as MAPK1, CSNK1D, and PRKD3 for up-regulated genes (Figure 7A), and MAPK14, ABL2, and INSR for down-regulated genes (Figure 7B) had connections with a large number of intermediate proteins and TFs (Table 5). The comparison of predicted kinases of up-regulated and down-regulated genes revealed that overall kinases were dissimilar barring ABL2 from up-regulated genes. Androgen receptor (AR) and nuclear factor-kB p65 (RelA) were observed to be a hub protein of down-regulated genes (via 48 direct and 3 indirect interactions) and up-regulated genes (via 80 direct and 3 indirect interactions), respectively. Therefore, the candidate TFs and their downstream target genes could play vital roles in the progression of autoimmune disease. These can be further explored as potential biomarkers for the diagnosis or treatment target.

**Table 4:** Transcription factors regulating the shared up- and down-regulated genes in EMS and MS identified using Expression2Kinases (X2K).

**Rank**	^*^TF	***P*-value**	**Z-score**	**^#^C-score**	**Target genes**
**TRANSCRIPTION FACTORS AS DOWN-REGULATION**					
1	SOX11_23321250	3.70E-06	−2.56	32.08	08 | ITGB8, PKD1L2, SALL1, SPAG9, MAP4, HIP1, MEIS1, SCD
2	AR_22383394	1.91E-06	−1.53	20.12	10 ±*ITGB*8, *FNDC*3*B, SLC*8*A*1, *PLEKHH*1, *UGT*8, *ANO*4, *CDH*1, *MAP*4, *CA*12, *MEIS*1
3	TRIM28_17542650	6.02E-01	−39.13	19.85	03 | MEIS1, SALL1, ERBB3
4	AHR_22903824	2.82E-04	−2.17	17.76	05 | FNDC3B, ZKSCAN1, ANO4, CLMN, MEIS1
5	POU5F1_16153702	1.90E-03	−2.61	16.38	04 | SALL1, CDH1, SPAG9, MEIS1
6	ARNT_22903824	2.08E-04	−1.93	16.35	06 | ZKSCAN1, ANO4, CLMN, FNDC3B, SALL1, MEIS1
7	MYCN_21190229	4.00E-03	−2.86	15.78	03 | CA12, CREB5, MEIS1
8	FOXA2_19822575	2.60E-06	−1.2	15.44	12 | EPB41L2, ZKSCAN1, FNDC3B, SLC8A1, HIP1, PPP2R5E, ZAK, CDH1, RCAN1, SPAG9, PTPN11, ERBB3
9	ELK1_19687146	1.01E-03	−2.15	14.86	05 | ITGB8, SMARCC1, NASP, PTPN11, MEIS1
10	TFAP2C_20629094	4.77E-04	−1.62	12.39	06 | EPB41L2, ZKSCAN1, CLMN, FNDC3B, HIP1, PPP2R5E
**TRANSCRIPTION FACTORS AS UP-REGULATION**					
1	POU3F2_20337985	1.76E-07	−1.88	29.29	11 | KCND2, AKAP12, SYNE2, COL11A1, LEPR, MKX, PDZRN4, SOD2, CHL1, GPM6A, NR4A2
2	BACH1_22875853	2.39E-06	−1.89	24.52	09 | LRRC2, COL11A1, LEPR, EGR1, AKAP12, SOD2, BCL6, SYNE2, NR4A2
3	STAT3_23295773	2.65E-09	−1.13	22.37	16 | KCND2, NAMPT, BCL6, SYNE2, BDNF, CHL1, AKAP12, SORBS2, RBMS3, COL11A1, LEPR, PDZRN4, CHRM3, ITPR1, NR4A2, PLCXD3
4	RELA_24523406	8.65E-06	−1.61	18.74	08 | BDNF, EGR1, KCNT2, PER1, SOD2, NAMPT, NR4A2, LTF
5	NR3C1_21868756	6.16E-05	−1.79	17.31	07 | AKAP12, SYTL2, FLRT3, FIGN, CHRM3, BCL6, GPM6A
6	TRIM28_17542650	6.48E-01	−38.96	16.9	03 | KCND2, BDNF, LRRC2
7	CTBP1_25329375	6.61E-05	−1.75	16.84	07 | LRRC2, KCNT2, PDZRN4, FLRT3, CHL1, CHRM3, SYNE2
8	CLOCK_20551151	5.17E-04	−2.21	16.76	04 | ITPR1, BCL6, EGR1, PER1
9	SMAD_19615063	5.94E-03	−3.25	16.66	02 | SYTL2, BDNF
10	AHR_22903824	3.90E-04	−2.1	16.48	05 | FIGN, LEPR, BCL6, MKX, NR4A2

* TF, transcription factor;

# *C-score, combine score of p-value and z-score, which is used to rank the identified TFs*.

**Table 5:** Protein kinase as up- and down-regulation responsible for phosphorylation of PPI at disease state of EMS and MS.

**Rank**	**Kinase**	***P*-value**	**Z-score**	**^#^C-score**	**Substrates**
**PROTEIN KINASE AS DOWN-REGULATION**					
1	MAPK14	3.03E-03	−2.40	13.91	05 | EPB41L2, RCAN1, SPAG9, SMARCC1, MAP4
2	ABL2	1.55E-03	−1.82	11.79	02 | PTPN11, ERBB3
3	INSR	7.73E-03	−2.06	10.02	03 | EPB41L2, ERBB3, PTPN11
4	ERBB4	4.56E-03	−1.82	9.83	02 | ERBB3, PTPN11
5	GSK3B	9.52E-03	−2.09	9.74	05 | CDH1, RCAN1, SMARCC1, MAP4, CLMN
6	PRKDC	1.56E-02	−1.85	7.70	03 | MAP4, NASP, SPAG9
7	PTK2B	1.24E-02	−1.65	7.24	02 | ERBB3, PTPN11
8	EGFR	1.95E-02	−1.83	7.21	03 | CDH1, ERBB3, PTPN11
9	MAPK8	2.59E-02	−1.77	6.45	03 | MAP4, NASP, SPAG9
10	PTK2	2.19E-02	−1.62	6.19	02| ERBB3, PTPN11
**PROTEIN KINASE AS UP-REGULATION**					
1	MAPK1	1.69E-02	−2.26	9.22	04 | KCND2, ITPR1, NR4A2, BCL6
2	CSNK1D	3.23E-02	−1.86	6.40	02 | AKAP12, PER1
3	PRKD3	2.61E-02	−1.47	5.38	01 | BCL6
4	WNK4	3.57E-02	−1.39	4.63	01 | BCL6
5	DDR1	5.78E-02	−1.52	4.34	01 | COL11A1
6	GRK6	5.47E-02	−1.40	4.08	01 | CHRM3
7	NTRK2	1.01E-01	−1.57	3.61	01 | BDNF
8	ABL2	7.64E-02	−1.39	3.57	01 | SORBS2
9	CAMK2D	1.13E-01	−1.38	3.01	01 | KCND2
10	CAMK2B	1.04E-01	−1.30	2.94	01 | KCND2

# *C-score, combine score of p-value and z-score, which is used to rank the identified kinase*.

## Discussion

**Figure 8:**
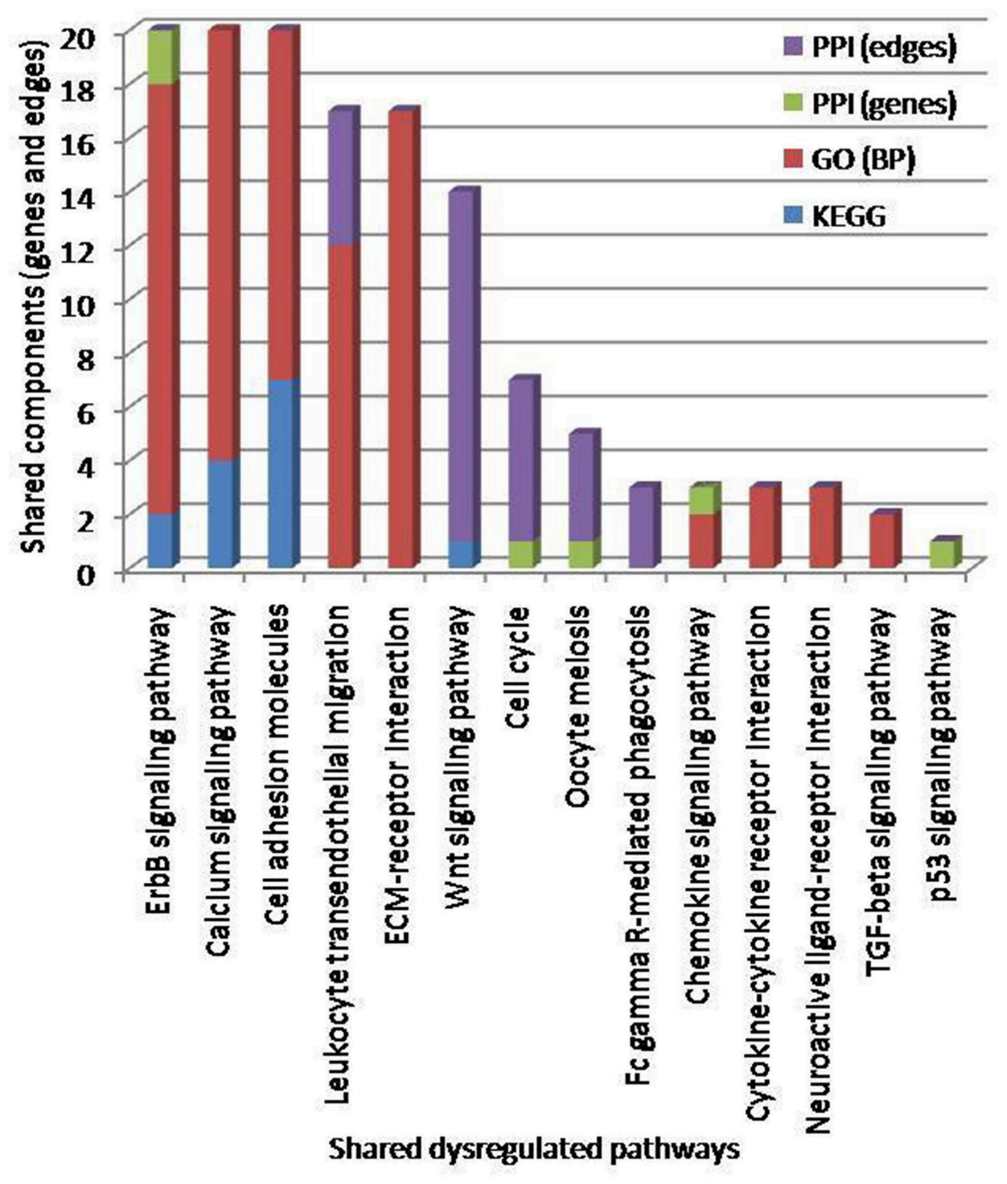
Pathways crosstalk through shared components (genes and edges). The number of identified crosstalk pathways by KEGG (blue), GO: BP (red), and PPI (green and violet) methods are indicated. The top three enriched pathways based on shared genes, edges and GO terms were erbB signaling pathways, calcium signaling pathways and cell adhesion molecules.

A number of studies have reported that women suffering from EMS are more prone to develop MS (Alviggi et al., 2006; Nielsen et al., 2011; Mormile and Vittori, 2014; Moghadasi and Salehizadeh, 2017). However, not much data is available to explain the immunological or defense mechanisms shared by these two autoimmune diseases. Similarly, the unique and shared molecular links accountable for failure or breakdown of self-tolerance, which lead to the development of MS in woman with EMS are also unclear. The present work has explored the publicly available microarray data for EMS vs. control and the MS vs. control cases and uncovered the shared molecular signatures which probably play a role in linking EMS with MS. Widely used enrichment analysis methods (Figure 1B) was adopted for the prediction of dysregulated pathways and establish subsequent possible crosstalk between EMS and MS. The enrichment analysis of differentially expressed genes (DEGs) by Kyoto Encyclopedia of Genes and Genomes (KEGG), Gene ontology (GO) and protein-protein interactions (PPI) divulged 46 disease-related pathways commonly disturbed in both diseases (Table S10 and Figure S4). The findings of disease-related pathways confined to the immune system, signal transduction, signaling molecules and interaction, and cell growth and death as the alteration in the suggested 14 pathways (Figure 8, Table 6) is known to be associated with shared risks of pathogenesis for both EMS and MS (Nielsen et al., 2011; Mormile and Vittori, 2014). The dysregulated pathways were mainly affected through common genes and edges associated with the inflammatory/immune responses. Downstream analysis revealed a number of crosstalks of dysfunctional pathways mediated through 23 overlapping unique genes that can interact with the signal transduction pathway (Table S11). Associated genes mainly corresponded to cell adhesion molecules (CADM3, CDH1, CLDN11, ITGB8, NCAM1, NEGR1, and NRXN1), neurotransmitters (CHRM3, GABRB2, GRIA2, GRIA3, and RXFP1), cytokine receptors (TGFBR2 and LEPR), and enzyme families (ERBB3, PLD1, PPP2R5E, and PTPN11). Other shared genes connected with cyclin (CCNB1), inositol trisphosphate receptor (ITPR1), laminin receptor (LAMB1), transport protein (SLC8A1), and adaptor protein (SHC3). These are well known immunomodulatory proteins or immune checkpoints that are negative regulators of the immune system. The up- and down-regulated expressions of 10 immunomodulatory proteins were detected commonly in both diseases (Table 7). A shared gene expression signature could form a common link between these two diseases. These results are consistent with preceding reports that excessive co-stimulation and/or insufficient co-inhibition of immunomodulatory molecules can result in a collapse of self-tolerance leading to the expansion of autoimmune diseases in humans (Ramsay, 2013; Zhang and Vignali, 2016). In addition, six interaction networks through overlapping edges of common dysregulated pathways of EMS and MS were also discovered (Table 3, Figure 6). This result suggested the probable associations of these two diseases through overlapping protein interactions. The identical GO biological processes related to inflammatory/immune responses have shown the functional overlap likely to infer the co-occurrence of EMS with MS (Figure 4). We have also identified a handful of hub genes PTPN11 (degree 15, betweenness 374.27), ERBB3 (degree 11, betweenness 198.32), and CDH1 (degree 10, betweenness 176) that are shared by these two disorders (Figure 5). This is consistent with the previous assumption that hub proteins are encoded by the essential genes and are associated with disease genes (Jeong et al., 2001). The shared pathways interacted in crosstalk systems were observed to be regulated by shared upstream regulators, leading to the activated or repressed immune response. POU3F2 (POU domain, class 3, transcription factor 2), BACH1 (breast cancer type 1 susceptibility protein; Igarashi et al., 2017), and STAT3 (signal transducer and activator of transcription 3; Kortylewski et al., 2005; Ho and Ivashkiv, 2006) were top 3 identified TFs from the shared up-regulated genes, leading to the activated immune response when down expressed in cells (Table 4, Figure 7A). Likewise, SOX11 (transcription factor SOX-11), AR (androgen receptor; Lai et al., 2012) and TRIM28 (transcription intermediary factor 1-beta; Ozato et al., 2008; Chikuma et al., 2012) were top 3 identified TFs from the shared down-regulated genes which lead to repression of the immune response when over-expressed in cells (Table 4, Figure 7B) (Schultz et al., 2002; Sripathy et al., 2006). This suggests the involvement of TFs in the association mechanism of both EMS and MS.

**Table 6:** Shared dysregulated pathways lead to endometriosis (EMS) and multiple sclerosis (MS) obtained through KEGG, GO and PPI methods.

**KEGG -ID**	**Dysregulated pathways**	**Classification**	^*^KEGG	**^#^GO**	**^$^PPI**		**^&^Hits**
					**Genes**	**Edges**	
hsa04012	ErbB signaling pathway	Signal transduction	2	16	2	0	20
hsa04020	Calcium signaling pathway	Signal transduction	4	16	0	0	20
hsa04514	Cell adhesion molecules	Signaling molecules and interaction	7	13	0	0	20
hsa04670	Leukocyte transendothelial migration	Immune system	0	12	0	5	17
hsa04512	ECM-receptor interaction	Signaling molecules and interaction	0	17	0	0	17
hsa04310	Wnt signaling pathway	Signal transduction	1	0	0	13	14
hsa04110	Cell cycle	Cell growth and death	0	0	1	6	7
hsa04114	Oocyte meiosis	Cell growth and death	0	0	1	4	5
hsa04666	Fc gamma R-mediated phagocytosis	Immune system	0	0	0	3	3
hsa04062	Chemokine signaling pathway	Immune system	0	2	1	0	3
hsa04060	Cytokine-cytokine receptor interaction	Signaling molecules and interaction	0	3	0	0	3
hsa04080	Neuroactive ligand-receptor interaction	Signaling molecules and interaction	0	3	0	0	3
hsa04350	TGF-beta signaling pathway	Signal transduction	0	2	0	0	2
hsa04115	p53 signaling pathway	Cell growth and death	0	0	1	0	1

* *KEGG, number of shared genes enriched within the common pathways of EMS and MS*.

# *GO, number of shared genes of biological process GO terms enriched within the common pathways of EMS and MS*.

$ *PPI, number of shared genes and edges associated with the protein-protein interactions within the common pathways of EMS and MS*.

& *Hits, the dysregulated pathways were ordered by the total number of hits*.

**Table 7:** Crosstalk interactions through shared overlapping genes or immunomodulatory proteins as probable linker in EMS and MS.

**Shared genes**	**Description**	**^#^EMS**	**^#^MS**	**Dysregulated shared pathways**	**Classification**
**UP-REGULATION IN BOTH EMS AND MS**					
NEGR1	Neuronal growth regulator 1	↑	↑	Cell adhesion molecules (CAMs)	Signaling molecules and interaction
LEPR	Leptin receptor	↑	↑	Cytokine-cytokine receptor interaction	Signaling molecules and interaction
CHRM3	Cholinergic receptor muscarinic 3	↑	↑	Neuroactive ligand-receptor interaction/// Calcium signaling pathway	Signaling molecules and interaction/// Signal transduction
ITPR1	Inositol 1,4,5-trisphosphate receptor type 1	↑	↑	Oocyte meiosis///Calcium signaling pathway	Cell growth and death/// Signal transduction
**DOWN-REGULATION IN BOTH EMS AND MS**					
SLC8A1	Solute carrier family 8 member A1	↓	↓	Calcium signaling pathway	Signal transduction
ERBB3	Erb-b2 receptor tyrosine kinase 3	↓	↓	Calcium signaling pathway///ErbB signaling pathway	Signal transduction
CDH1	Cadherin 1	↓	↓	Cell adhesion molecules (CAMs)	Signaling molecules and interaction
ITGB8	Integrin subunit beta 8	↓	↓	Cell adhesion molecules (CAMs)///ECM-receptor interaction	Signaling molecules and interaction
PTPN11	Protein tyrosine phosphatase, non-receptor type 11	↓	↓	Leukocyte transendothelial migration	Immune system
PPP2R5E	Protein phosphatase 2 regulatory subunit B'epsilon	↓	↓	Oocyte meiosis	Cell growth and death

*# Up arrow, denotes to upregulation of genes*.

*# Down arrow, denotes to downregulation of genes*.

### Immune modulation through dysregulation of cytokine

The systemic immune alteration due to dysregulation of cytokine or chemokines production is well known to be associated with the pathogenesis of EMS (Cakmak et al., 2009; Herington et al., 2011) and MS (Sellebjerg et al., 2009; Hasheminia et al., 2015; Becher et al., 2017). The sites associated with inflammation attract leukocytes through a group of low molecular weight proteins, the chemoattractant cytokines or chemokines. The role of CC chemokine receptor 5 (CCR5) and its CC chemokine ligand 3 (CCL3) has been suggested in Th1-type inflammatory/immune response (Bleul et al., 1997; Wu et al., 1997; Bonecchi et al., 1998; Patterson et al., 1999; Nansen et al., 2002). Reduced expression of CCL3 in MS was observed, suggesting a concomitant reduction of CCR5 that might contribute in the development of inflammatory demyelination (Banisor et al., 2005). Interleukin 8 (IL-8/CXCL8) is a chemoattractant for monocytes and neutrophils, which is involved in the attraction and infiltration of leukocytes at the site of inflammation. We observed increased expression of CXCL8 in MS patients, which is generally not perceived in normal patients (Lund et al., 2004; Bartosik-Psujek and Stelmasiak, 2005). We also detected elevated levels of chemokine CXCL13 and its receptor CXCR5 which involve the maintenance of pathogenic B cells in autoimmune diseases like MS (Finch et al., 2013). Likewise, the study of genetic polymorphisms of chemokines elucidates an association between rs2812378 and C-C motif chemokine ligand 21 (CCL21) in the advanced stages of endometriosis (Bellelis et al., 2015). These findings signify that chemokines and their receptors are important for the development and maintenance of innate and adaptive immunity (Esche et al., 2005; Raman et al., 2011). The results also suggest that altered expression of β-chemokines are involved in similar biological processes or function in both EMS and MS (Table S7B). In addition, crosstalk analysis exposed that the stimulation of cytokine-cytokine receptor interaction pathway in EMS, up-regulates a gene LEPR or activates a protein leptin receptor that is also a member of the same pathway in MS. Therefore, the down-stream response of the suggested pathways in both EMS and MS might be regulated by the same activated TFs, resulting in the up-regulated expression of LEPR genes in MS. Previous reports have confirmed that leptin (acts via leptin receptor) is negatively correlated with the production of regulatory T cells and hence associated with immune deficiency (Farooqi et al., 2002). Therefore, the elevated expression of leptin/leptin receptor in EMS prompts immune deficiency which may induce MS.

### Immune modulation through dysregulation of adhesion molecules

Our evaluation indicated that patients suffering from EMS exhibited an upregulated expression of neuronal growth regulator 1 (NEGR1) with downregulated expression of Catherine 1 (CDH1) and integrin subunit beta 8 (ITGB8). A similar trend for these proteins was also observed for MS. The stimulation of cell adhesion molecules (CAMs) pathway in EMS, up-regulates a gene NEGR1 or turns on a protein neuronal growth regulator 1 that is also an element of the identical pathway in MS. Therefore, the down-movement reaction of these pathways in EMS and MS is probably regulated through the same activated TFs, resulting in concomitant up-regulation of NEGR1 genes in MS in patients with EMS. The overexpression of NEGR1 gene prevents synaptogenesis leading to autoimmune or neurodegenerative diseases. These results are consistent with the previous hypothesis that the precise expression of neuronal growth regulator 1 is critical for the neurite outgrowth in the brain, while the dysregulated expression of negr1 gene is known to play a key function in inhibiting neurite outgrowth and synapse formation (Gil et al., 1998; Schäfer et al., 2005; Hashimoto et al., 2009; Pischedda et al., 2014; Sanz et al., 2015). The inhibition of cell adhesion molecule pathway in EMS inhibits the genes CDH1/cadherin-1 and ITGB8/integrin subunit beta 8 in the pathway which co-occurs in MS. Activated TFs are expected to regulate this down-flow reaction resulting in the down-regulation of CDH1 and ITGB8 genes in MS. The ligand αEβ7 integrin, expressed in several subsets of lymphocytes acts via its receptor cadherin-1 or E-cadherin which is expressed in epithelial cells. This interaction results in adhesion of lymphocytes to epithelial cells, probably important for T cell homing to the intestinal sites (Agace et al., 2000). Our results demonstrated the down-regulation of E-cadherin, which is known to increase cellular motility by weakening cellular adhesion within a tissue. The findings point toward the increased risk of altered intestinal immune reactions and tissue injuries connected with inflammatory diseases, including autoimmune diseases (Yoshimoto et al., 2014). This suggests that αEβ7 could be used as a potential therapeutic target for inflammatory diseases. Likewise, the cytokine transforming growth factor-beta (TGF-β) acts as an immune suppressor during homeostasis, infection and disease (Yoshimura and Muto, 2011; Worthington et al., 2012). The down-regulation of integrin subunit beta 8 (ITGB8) was observed, suggesting the interruption in alpha-V/beta-8-mediated TGF-β activation through dendritic cells which is vital for preventing immune disorder (Travis et al., 2007).

### Immune modulation through dysregulation of neurotransmitters

The stimulation of neuroactive ligand-receptor interaction pathway as well as calcium signaling pathway in EMS was observed. This up-regulates a gene CHRM3 or initiates a protein cholinergic muscarinic receptor subtype 3 in EMS which coexists in MS. Consequently, the CHRM3 gene was also seen to be up-regulated in MS. CHRM3 signaling modulates the activation, recruitment and differentiation of progenitor cells or oligodendrocytes precursor cells (OPCs), essential for the remyelination in the central nervous system (Abiraman et al., 2015). The crosstalk analysis disclosed the dysregulation of OPCs by the overexpression of CHRM3 gene, which inhibits remyelination and enhances the risk of MS in patients suffering from EMS.

### Immune modulation through dysregulation of InsP3R and transport protein

Crosstalk research has proven that the stimulation of calcium signaling pathway in EMS, up-regulates the gene ITPR1. Since an identical pathway is also present in MS, the increased ITPR1 levels can induce MS. The protein Inositol 1,4,5-trisphosphate (InsP3/Ins3P/IP3) is an essential signal transduction element of the calcium (Ca^2+^) signaling pathway involved in the regulation of cellular activities (Berridge, 1993; Hirota et al., 2003). Overexpression of ITPR1 gene results in calcium dysregulation accentuating the risk of autoimmune diseases (Zhuang et al., 2015). Our analysis suggested that the inhibition of the calcium signaling pathway in EMS, down-regulates the same pathway in MS. Hence, the expression of Solute carrier family 11A member 1 (SLC11A1) genes is decreased. SLC11A1 possess an immunomodulatory role in manipulating macrophage activation status (M1/M2) and Th1/Th2 biases. The transcriptional repression of SLC11A1 gene leads to cell proliferation and survival resulting in cancer and autoimmunity (Awomoyi, 2007). The reduced level of SLC11A1 genes is linked with the overexpression of cell proliferation in EMS patients. This increases the probability of developing MS in patients with EMS.

### Immune modulation through dysregulation of enzyme families

The inhibition of calcium signaling/ErbB signaling, leukocyte transendothelial migration and oocyte meiosis pathways as seen by our analysis in EMS, down-regulates the genes ERBB3 (Erb-b2 receptor tyrosine kinase 3), PTPN11 (tyrosine-protein phosphatase non-receptor type 11), and PPP2R5E (phosphatase 2 regulatory subunit B'epsilon), respectively. Common TFs regulate them in MS resulting in their synergistic decreased expression. The reduced expression of ERBB3 gene could contribute to insufficient remyelination associated with the expansion of neurodegenerative diseases like MS and Alzheimer's (Bublil and Yarden, 2007). These results are consistent with the previous study that neuregulin-1/ErbB signaling plays a crucial role in of OPC proliferation, oligodendrocyte differentiation and remyelination in the central nervous system (Vartanian et al., 1997; Canoll et al., 1999; Flores et al., 2000; Calaora et al., 2001). Likewise, protein tyrosine phosphatases (PTPs) act as negative regulators of the immune response in normal and pathophysiological conditions by controlling the activation/inhibition of lymphocytes. The observed reduction in the level of PTPs could be associated with abnormal lymphocyte function in both EMS and MS (Dolton et al., 2006; Rhee and Veillette, 2012). Similarly, the downregulated expression of PPP2R5E genes, exhibit the overexpression of regulatory T cells, ensuing the risk of autoimmunity. This is consistent with reports that protein phosphatase 2A (PP2A) is critical for regulatory T cells to function, for in their absence they no longer possess the ability to suppress effector T cells and thus fail to protect against autoimmunity (Apostolidis et al., 2016).

## Conclusions

The microarray gene expression data from healthy individuals and patients suffering from either of the two related autoimmune disorders, endometriosis or MS, from the GEO database was examined through KEGG, GO, and PPI methods. We identified shared differentially expressed genes in these two related diseases which were involved in common disease-related pathways. We also identified the links between these two diseases by analyzing their overlapping disease genes and edges through protein-protein interactions. The maximum number of shared genes and edges in shared dysregulated pathways was associated with the inflammatory/immune responses. The observed dysfunctional pathways mediated through an overlapping immunomodulatory protein are anticipated to act as probable linkers in EMS and MS. The enrichment analysis of GO terms clearly showcased the functional overlap of biological processes inferring the co-occurrence of EMS with MS. This analysis also suggested that the identified disease genes not shared through protein-protein interaction might play vital roles in the same or related functions to complete the molecular links. The identified common molecular signatures (such as genes, pathways, transcription factors, and protein kinases) can be further explored as novel targets/biomarkers for the simultaneous treatment of EMS and MS. The findings from this study increase our understanding of the molecular mechanisms affecting both EMS and MS and suggest an interconnection between the two diseases.

## Author contributions

AK initiated the research and performed the downstream analyses. PK conceived the idea of the study and designed the experiments. SS assisted in the analysis. AK, PK, and TPS interpreted the results and drafted the manuscript. All authors have read and approved the manuscript for publication.

### Conflict of interest statement

The authors declare that the research was conducted in the absence of any commercial or financial relationships that could be construed as a potential conflict of interest.

## Abbreviations

BP: biological process
BiNGO: Biological Networks Gene Ontology tool
DAVID: database for annotation, visualization and integrated discovery
DEG: differentially expressed genes
EMS: endometriosis
FDR: false discovery rate
GEO: gene expression omnibus
GO: gene ontology
JI: Jaccard Index
KEGG: Kyoto encyclopedia of genes and genomes
MS: multiple sclerosis
NCBI: national center for biotechnology information
OC: overlap coefficient
PPI: protein-protein interaction.
