# Supplementary material for: Identification of Shared Molecular Signatures Indicate the Susceptibility of Endometriosis to Multiple Sclerosis
Source: Front Genet. 2018 Feb 16;9:42. doi: 10.3389/fgene.2018.00042 (PMC5820528; doi:10.3389/fgene.2018.00042)
Supplement: Supplementary file 1 [file Image1.PDF]

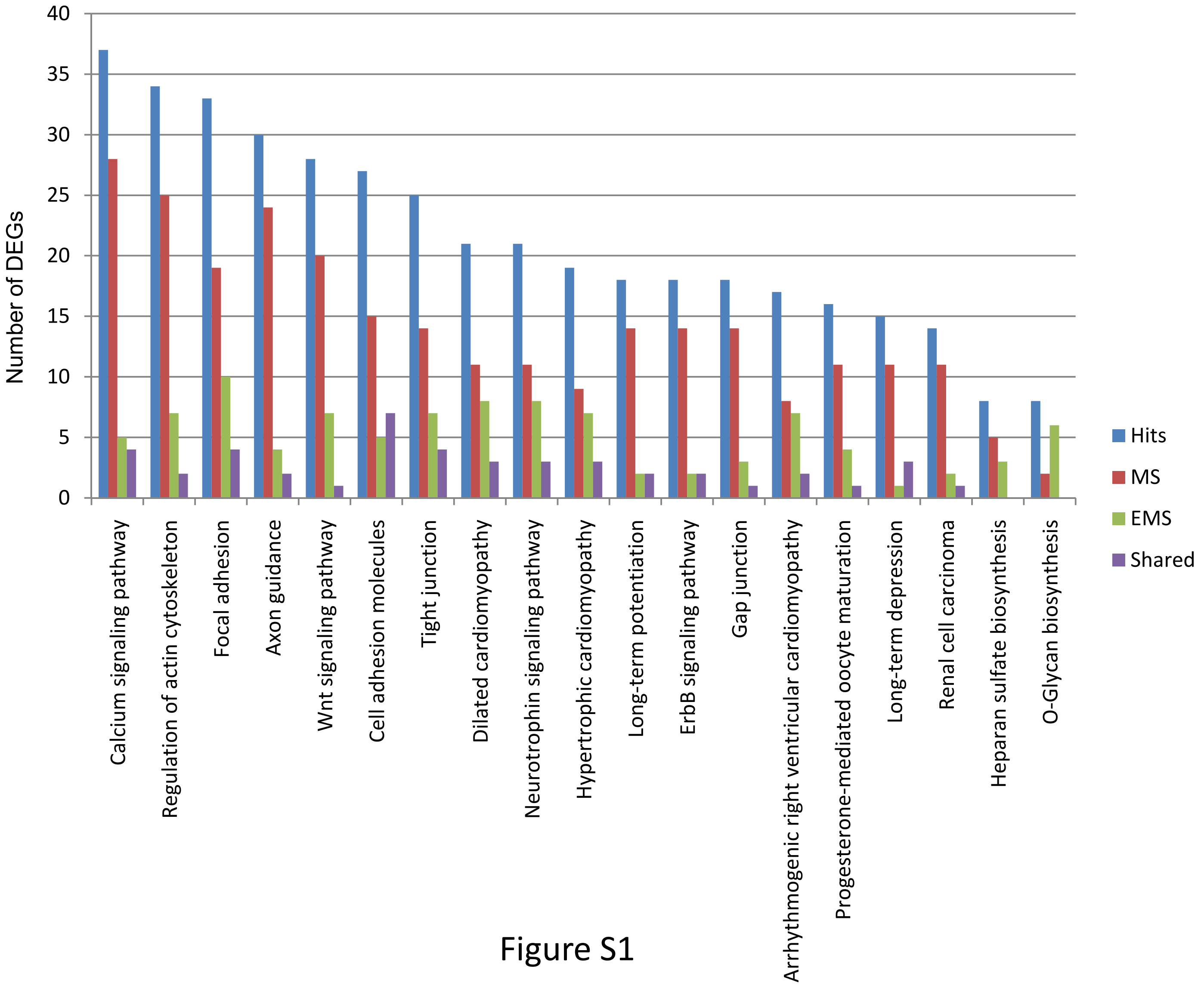

Figure S1

Over-represented pathways enriched with differentially expressed genes. the column chart shows the distribution of EMS (24%), MS (65%) and shared (11%) DEGs enriched in over-represented pathways. We observed a maximum number of enriched genes, i.e., 37, 34 and 33 for calcium signaling pathway (hsa04020), regulation of actin cytoskeleton (hsa04810) and focal adhesion (hsa04510), respectively.

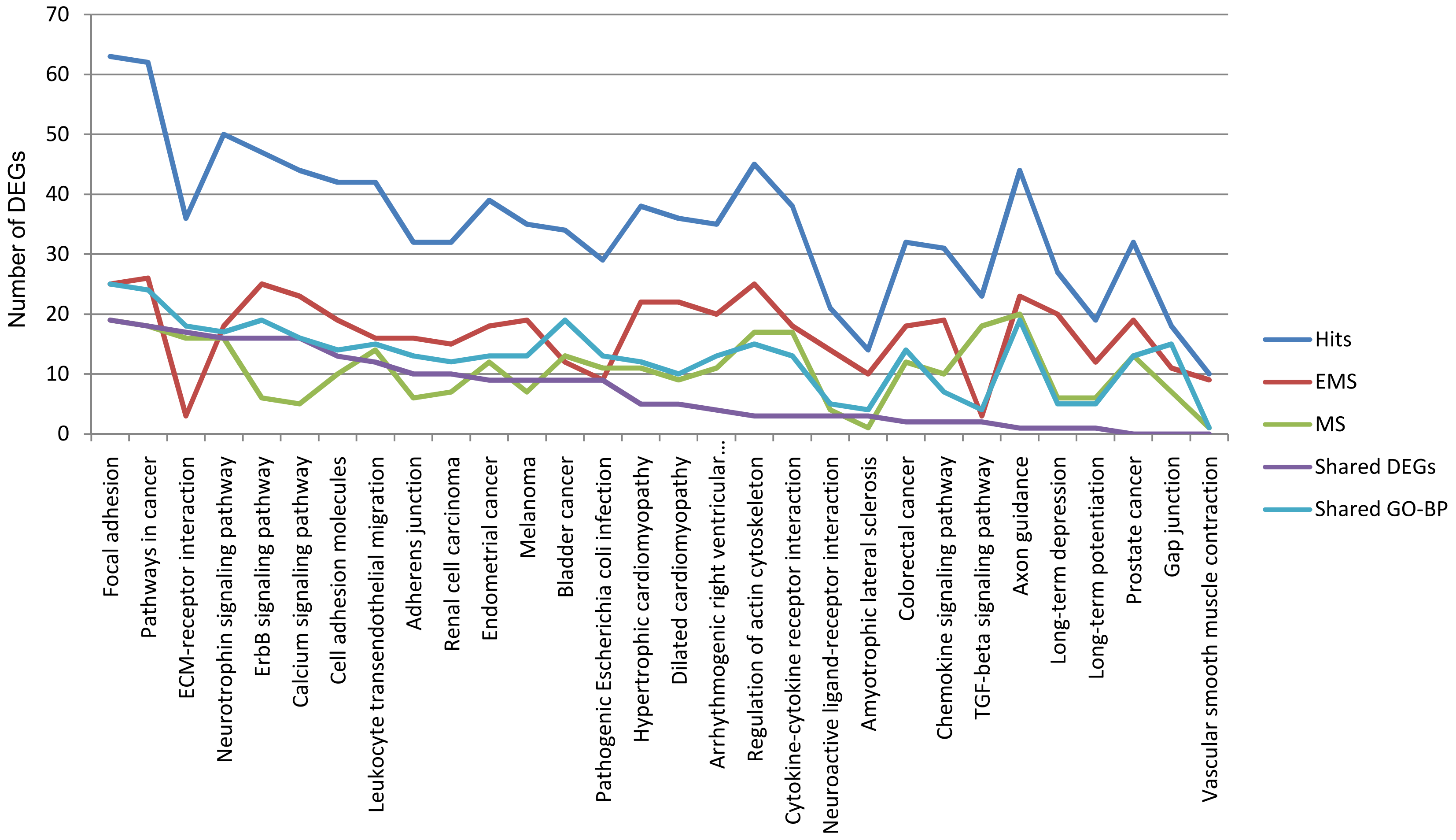

Figure S2

Over-represented pathways with differentially expressed genes and GO terms . The graph shows the over-represented pathways enriched with the number of DEGs and GO terms of EMS and MS. The findings uncovered 28 over-represented GO terms enclosed 511 DEGs.

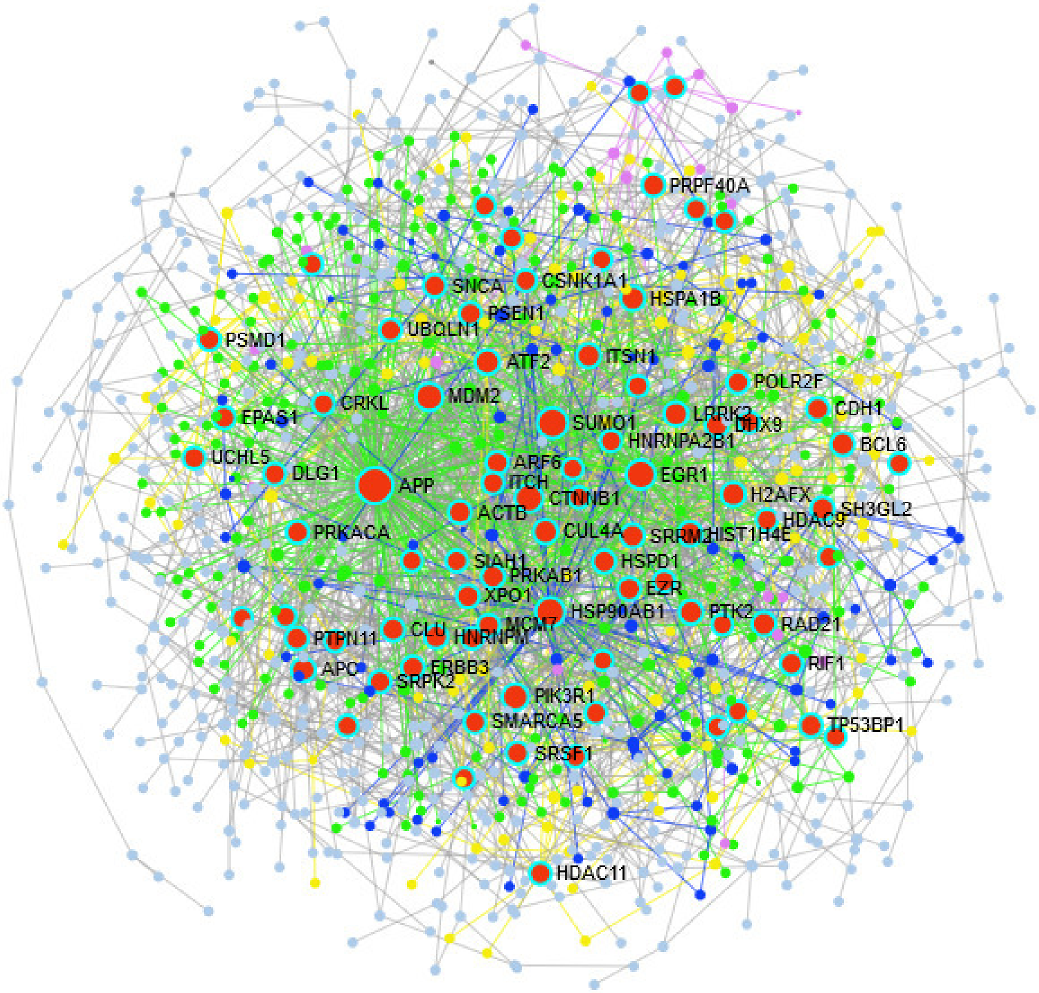

Figure S3

Protein–protein interactions network of DEGs. Represents the associations of 1029 nodes and 2136 edges in the constructed network. The EMS, MS and shared DEGs in the PPI network modules were denoted by the color yellow, green and blue, respectively. The hub genes with high degree and high betweenness were denoted with red color.

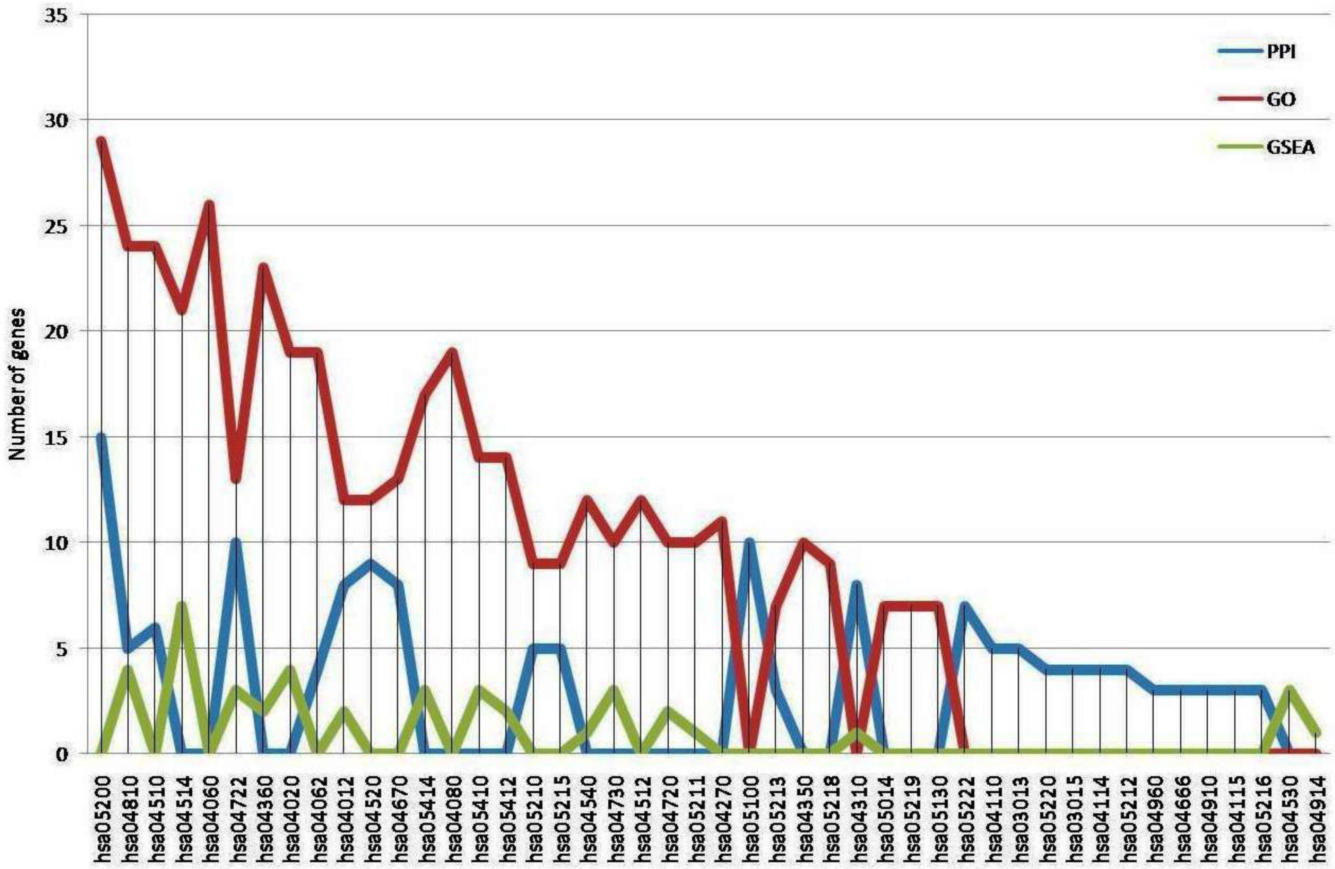

hsa05200 Pathways in cancer
hsa04810 Regulation of actin cytoskeleton
hsa04510 Focal adhesion
hsa04514 Cell adhesion molecules (CAMs)
hsa04060 Cytokine-cytokine receptor interaction
hsa04722 Neurotrophin signaling pathway
hsa04360 Axon guidance
hsa04020 Calcium signaling pathway
hsa04062 Chemokine signaling pathway
hsa04012 ErbB signaling pathway
hsa04520 Adherens junction
hsa04670 Leukocyte transendothelial migration
hsa05414 Dilated cardiomyopathy
hsa04080 Neuroactive ligand-receptor interaction
hsa05410 Hypertrophic cardiomyopathy (HCM)
hsa05412 Arrhythmogenic right ventricular cardiomyopathy (ARVC)
hsa05210 Colorectal cancer
hsa05215 Prostate cancer
hsa04540 Gap junction
hsa04730 Long-term depression
hsa04512 ECM-receptor interaction
hsa04720 Long-term potentiation
hsa05211 Renal cell carcinoma
hsa04270 Vascular smooth muscle contraction
hsa05100 Bacterial invasion of epithelial cells
hsa05213 Endometrial cancer
hsa04350 TGF-beta signaling pathway
hsa05218 Melanoma
hsa04310 Wnt signaling pathway
hsa05014 Amyotrophic lateral sclerosis (ALS)
hsa05219 Bladder cancer
hsa05130 Pathogenic Escherichia coli infection
hsa05222 Small cell lung cancer
hsa04110 Cell cycle
hsa03013 RNA transport
hsa05220 Chronic myeloid leukemia
hsa03015 mRNA surveillance pathway
hsa04114 Oocyte meiosis
hsa05212 Pancreatic cancer
hsa04960 Aldosterone-regulated sodium reabsorption
hsa04666 Fc gamma R-mediated phagocytosis
hsa04910 Insulin signaling pathway
hsa04115 p53 signaling pathway
hsa05216 Thyroid cancer
hsa04530 Tight junction
hsa04914 Progesterone-mediated oocyte maturation

Figure S4

Differentially expressed genes / pathways retrieved from KEGG, GO and PPI methods divulged 46 disease specific key pathways commonly disturbed in both two diseases.
